# Supplementary material for: How conflict shapes evolution in poeciliid fishes
Source: Nat Commun. 2019 Jul 26;10:3335. doi: 10.1038/s41467-019-11307-5 (PMC6659687; doi:10.1038/s41467-019-11307-5)
Supplement: Supplementary file 1 — Supplementary Information [file 41467_2019_11307_MOESM1_ESM.pdf]

Supplementary Information

How conflict shapes evolution in poeciliid fishes

Furness et al.

Supplementary Note 1: Ancestral state reconstruction of female reproductive mode (p. 2 – 7)
Supplementary Tables 1-2, Supplementary Figures 1-5

Supplementary Note 2: Ancestral state reconstruction of male attributes (p. 8 - 13)
Supplementary Tables 3-4, Supplementary Figures 6-10

Supplementary Note 3: Joint evolution of male and female traits (p. 14 - 25)
Supplementary Tables 5-8, Supplementary Figures 11-18

Supplementary Note 4: Speciation rate (p. 26 – 32)
Supplementary Tables 9-13, Supplementary Figure 19

Supplementary Note 5: Complementary analyses treating MI as a continuous, rather than dichotomous, variable (p. 33 – 39)
Supplementary Tables 14-15, Supplementary Figures 20-22

Supplementary References (p. 40)

Supplementary Note 1: Ancestral state reconstruction of reproductive mode

This section provides additional details regarding the ancestral state reconstruction of female reproductive mode (i.e. internal fertilization, live bearing, placentotrophy, and superfetation). First, we show a comparison of transition rate models for each character (Supplementary Table 1), and a summary of the predicted ancestral female reproductive mode of the Poeciliinae (Supplementary Table 2). We then show phylogenetic trees illustrating the maximum likelihood ancestral state reconstructions on all in-group and out-group taxa (Supplementary Figures 1-4) and mirrored trees illustrating the evolution of superfetation and placentotrophy in the Poeciliinae (Supplementary Figure 5).

Character	Taxa	Model	d.f.	log-likelihood	AIC
Internal fertilization (binary)	289	ARD	2	-19.504	43.007
		ER	1	-19.525	41.050
Livebearing (binary)	291	ARD	2	-23.100	50.201
		ER	1	-23.250	48.500
Placentotrophy (binary)	269	ARD	2	-55.487	114.974
		ER	1	-55.616	113.232
Superfetation (binary)	269	ARD	2	-30.055	64.110
		ER	1	-30.762	63.525

Supplementary Table 1. Comparison between equal rate (ER) and all rate different (ARD) transition rate models. For each female binary trait we esimated the likelihood of each Markov model using the "ace" command in the R package ape[1]. We then compared the fit of the models using AIC. For each binary trait the best-supported model, with the lowest AIC, was the equal rate model. The best-supported model was subsequently used in likelihood ancestral state reconstructions (Supplementary Table 2, Supplementary Figures 1-4).

Character	Character state	Proportional likelihood	Parsimony
Internal fertilization	Absent	0.00	0
	Present	1.00	1
Livebearing	Absent	0.13	0/1
	Present	0.87	0/1
Placentotrophy	Absent	0.96	0/1
	Present	0.04	0/1
Superfetation	Absent	1.00	1
	Present	0.00	0

Supplementary Table 2. Ancestral female reproductive mode of the Poeciliinae inferred using maximum likelihood and parsimony. Maximum likelihood reconstructions were performed in the R package ape[1], and parsimony reconstructions in Mesquite[2].

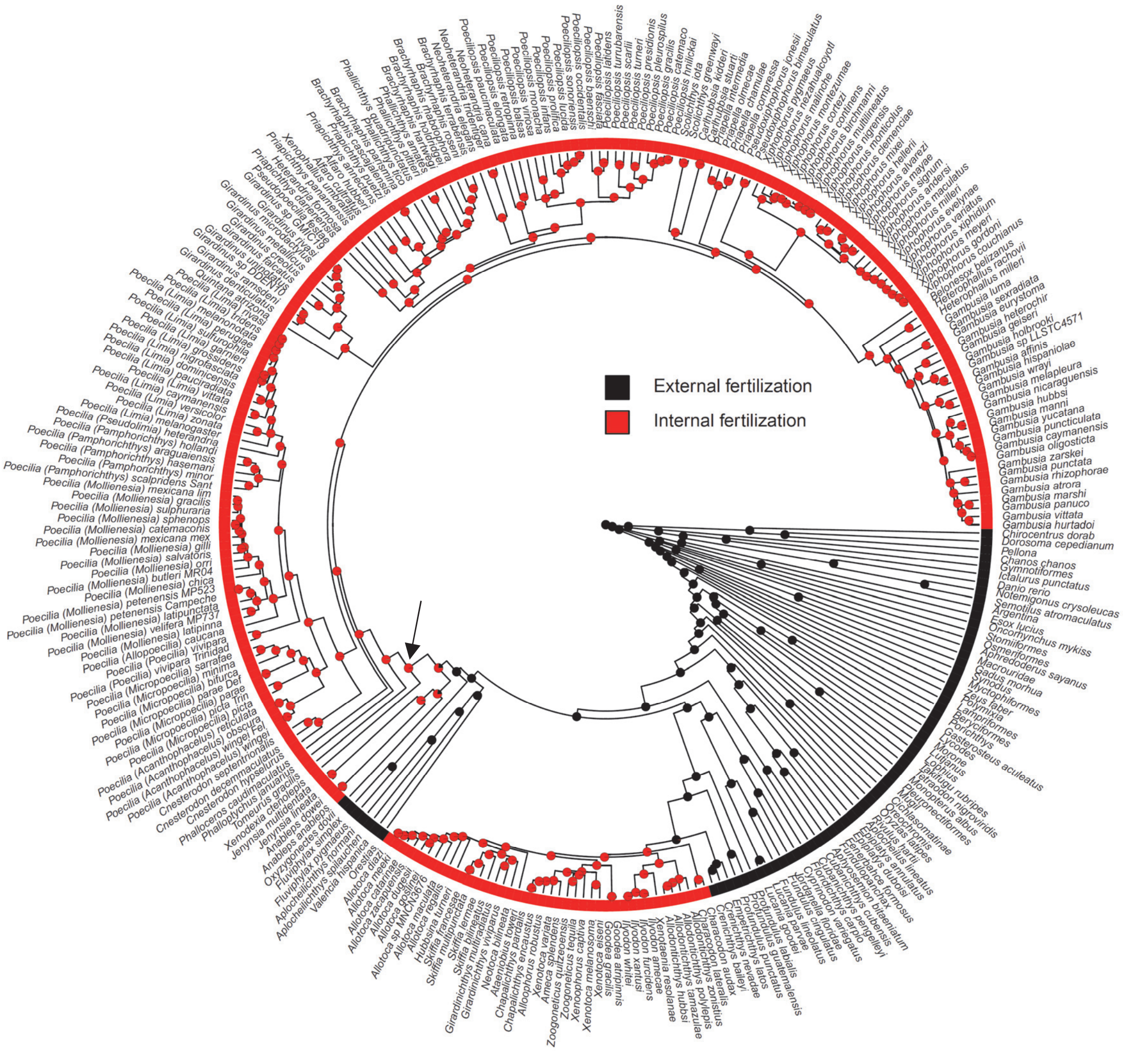

Supplementary Figure 1. Maximum likelihood ancestral state reconstruction of fertilization (external / internal) under equal rates model. The black arrow indicates the origin of the Poeciliinae.

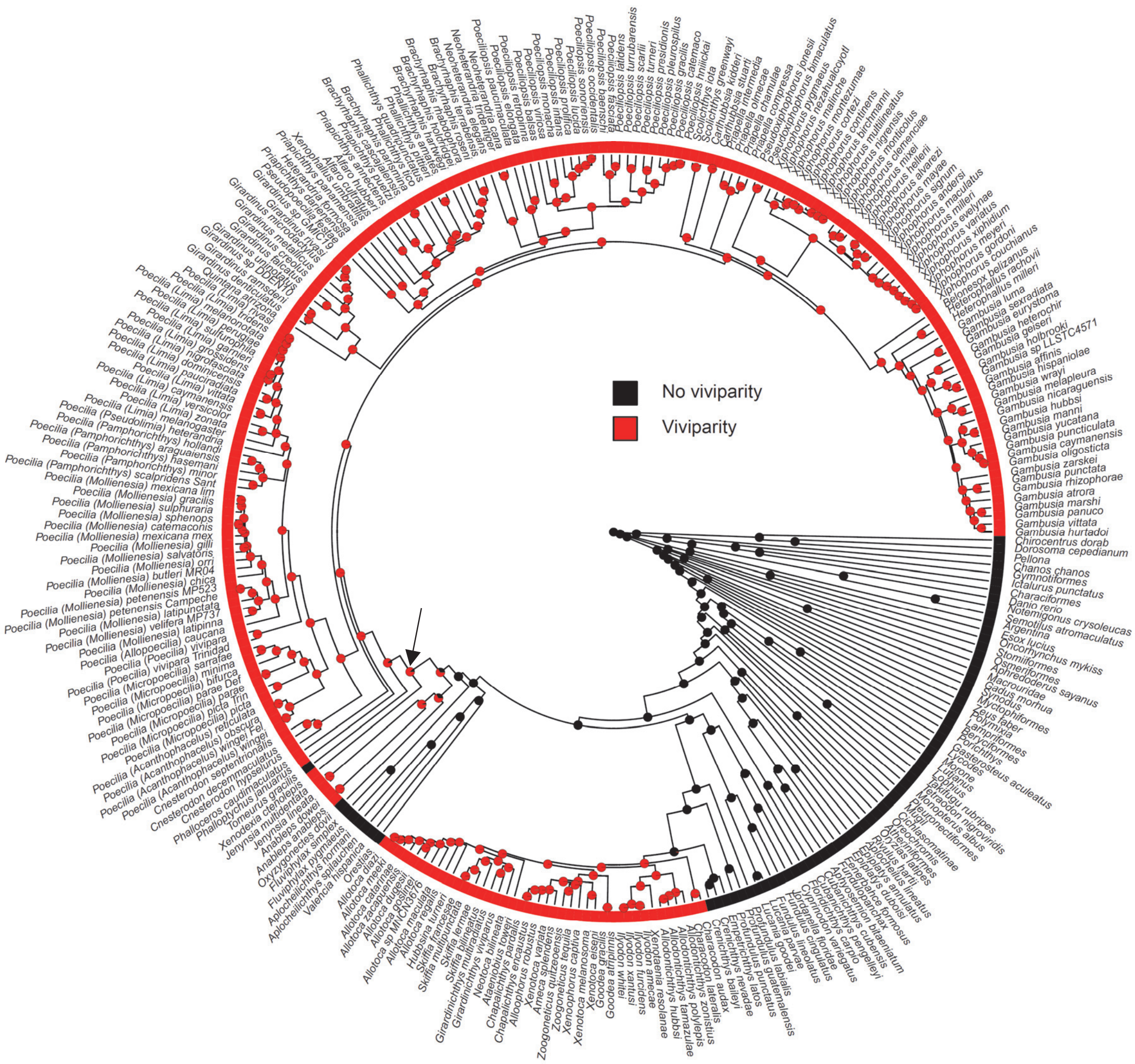

Supplementary Figure 2. Maximum likelihood ancestral state reconstruction of viviparity (absent / present) under equal rates model. The black arrow indicates the origin of the Poeciliinae.

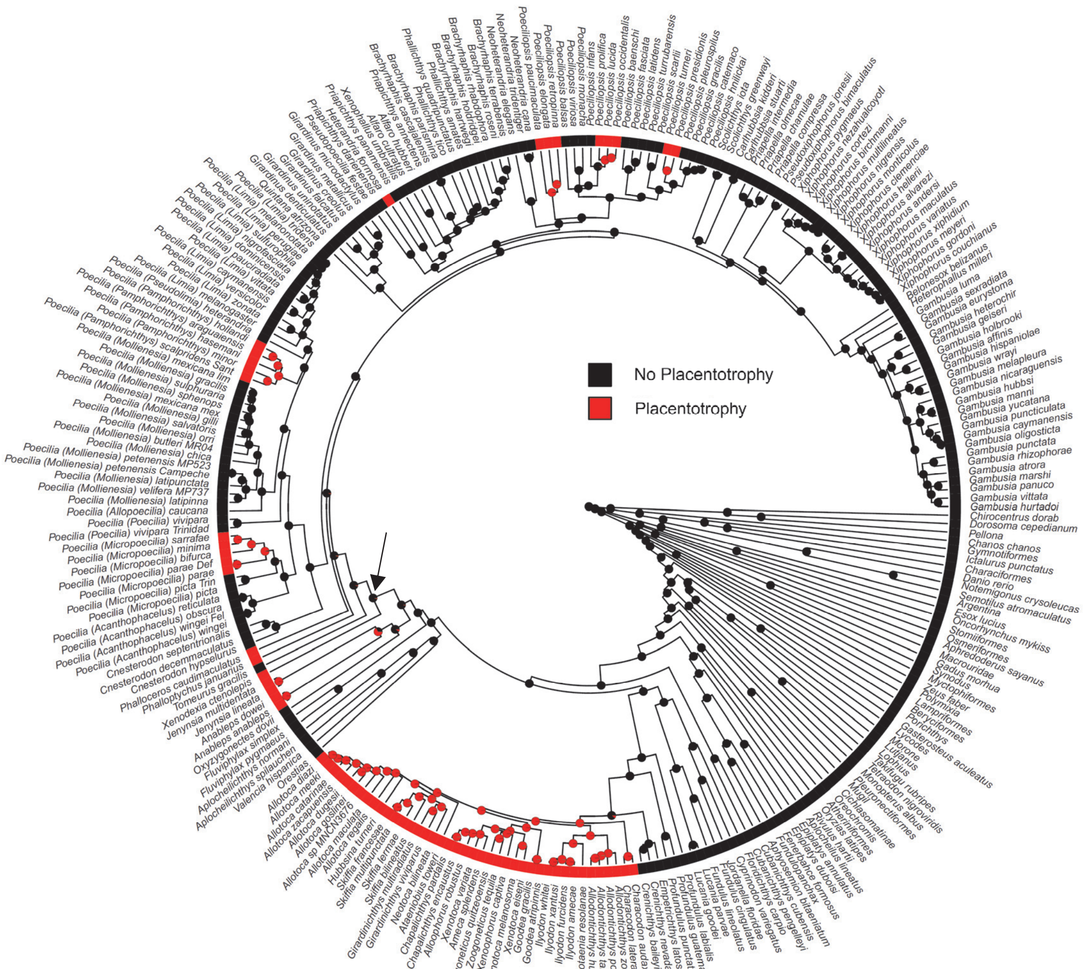

Supplementary Figure 3. Maximum likelihood ancestral state reconstruction of placentotrophy (absent / present) under equal rates model. The black arrow indicates the origin of the Poeciliinae.

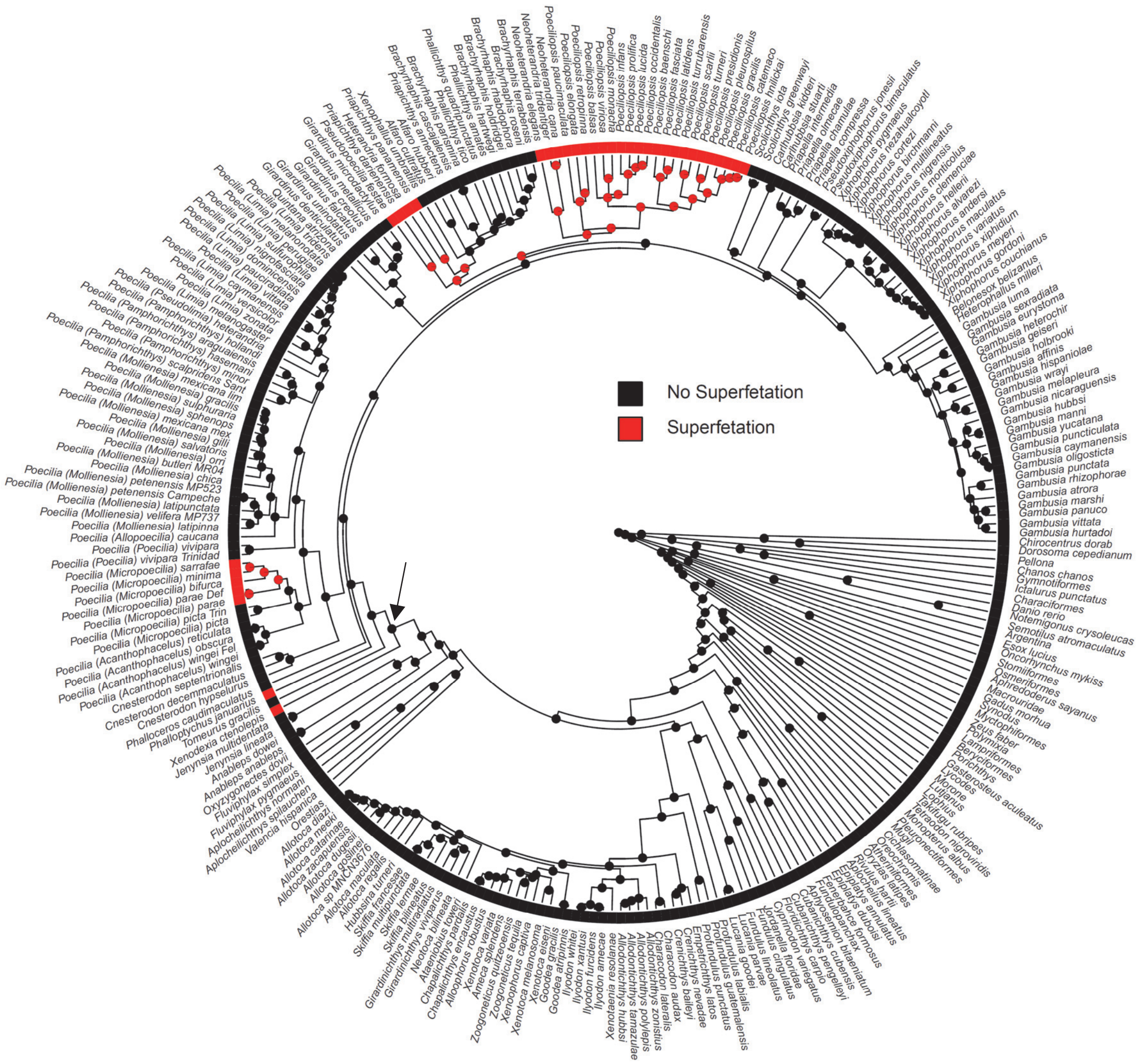

Supplementary Figure 4. Maximum likelihood ancestral state reconstruction of superfetation (absent / present) under equal rates model. The black arrow indicates the origin of the Poeciliinae.

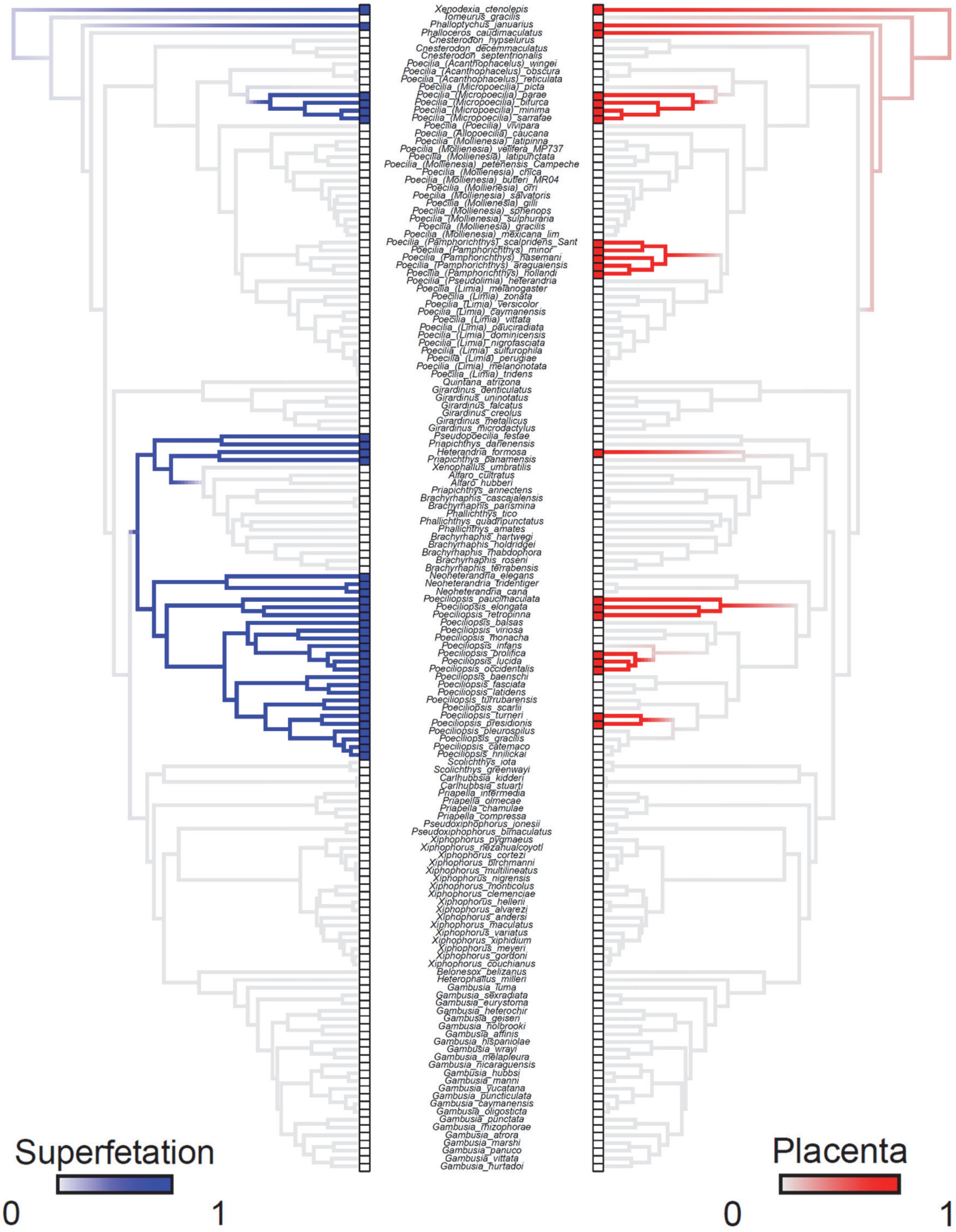

Supplementary Figure 5. Mirrored trees illustrating correlated evolution between superfetation and the placenta in the Poeciliinae. Ancestral state reconstructions of superfetation and the placenta by stochastic character mapping. Phylogeny restricted to species with both superfetation and placentation data (n=150). Branch colors represent posterior probability densities of edge states based on 1000 stochastic character maps of each reconstruction.

Supplementary Note 2: Ancestral state reconstruction of male attributes

This section provides additional details regarding the ancestral state reconstruction of male attributes (i.e. courtship, dichromatism, ornamentation, gonopodium length, and sexual dimorphism index). First, we show a comparison of transition rate models for each character (Supplementary Table 3), and a summary of the predicted ancestral male attributes of the Poeciliinae (Supplementary Table 4). We then show phylogenetic trees illustrating the maximum likelihood ancestral state reconstructions of each male trait (Supplementary Figures 6-10).

Character	Taxa	Model	d.f.	log-likelihood	AIC
Courtship (binary)	79	ARD	2	-47.992	99.983
		ER	1	-49.813	101.626
Dichromatism (binary)	94	ARD	2	-61.134	126.269
		ER	1	-61.251	124.501
Ornamentation (binary)	94	ARD	2	-26.135	56.270
		ER	1	-29.900	61.799
Gonopodium length (binary)	92	ARD	2	-38.025	80.050
		ER	1	-38.629	79.257
Sexual dimorphism index SL (binary)	90	ARD	2	-53.716	111.431
		ER	1	-54.538	111.076

Supplementary Table 3. Comparison between equal rate (ER) and all rate different (ARD) transition rate models. For each male binary trait we esimated the likelihood of each Markov model using the "ace" command in the R package ape[1]. We then compared the fit of the models using AIC. For each binary trait, save ornamentation, the best-supported model, with the lowest AIC or difference in AIC<2, was the equal rate model. The best-supported model was subsequently used in likelihood ancestral state reconstructions (Supplementary Table 4, Supplementary Figures 6-10).

Character	Character state	Proportional likelihood	Parsimony
Courtship	Absent	0.59	1
	Present	0.41	0
Dichromatism	Absent	0.53	1
	Present	0.47	0
Ornamentation	Absent	0.55	1
	Present	0.45	0
Gonopodium length	Short	0.08	0
	Long	0.92	1
Sexual dimorphism index (standard length)	Low	0.41	0
	High	0.59	1

Supplementary Table 4. Ancestral male attributes of the Poeciliinae inferred using maximum likelihood and parsimony. Maximum likelihood reconstructions were performed in the R package ape[1], and parsimony reconstructions in Mesquite[2].

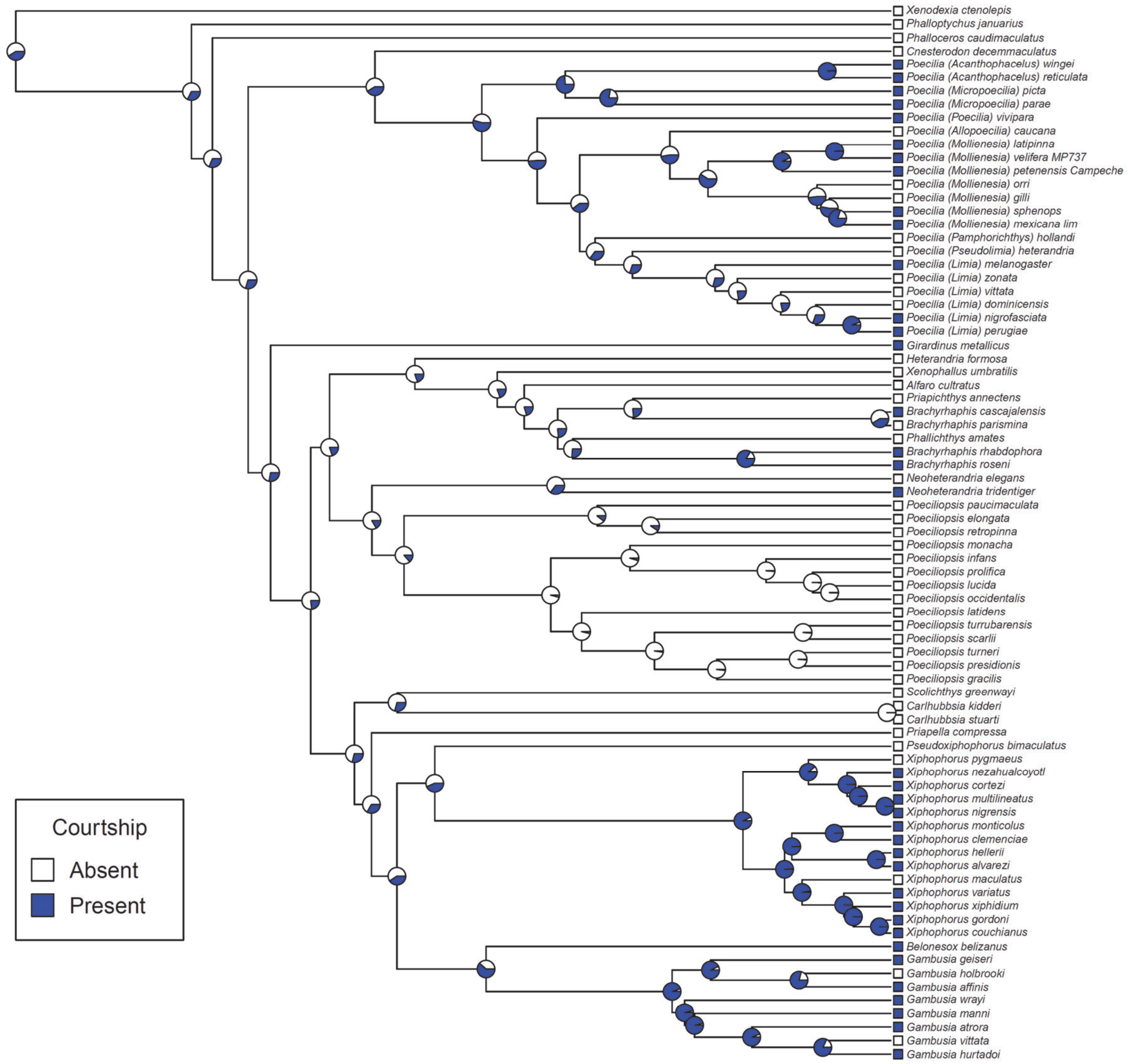

Supplementary Figure 6. Maximum likelihood ancestral state reconstruction of courtship (absent / present) under equal rates model.

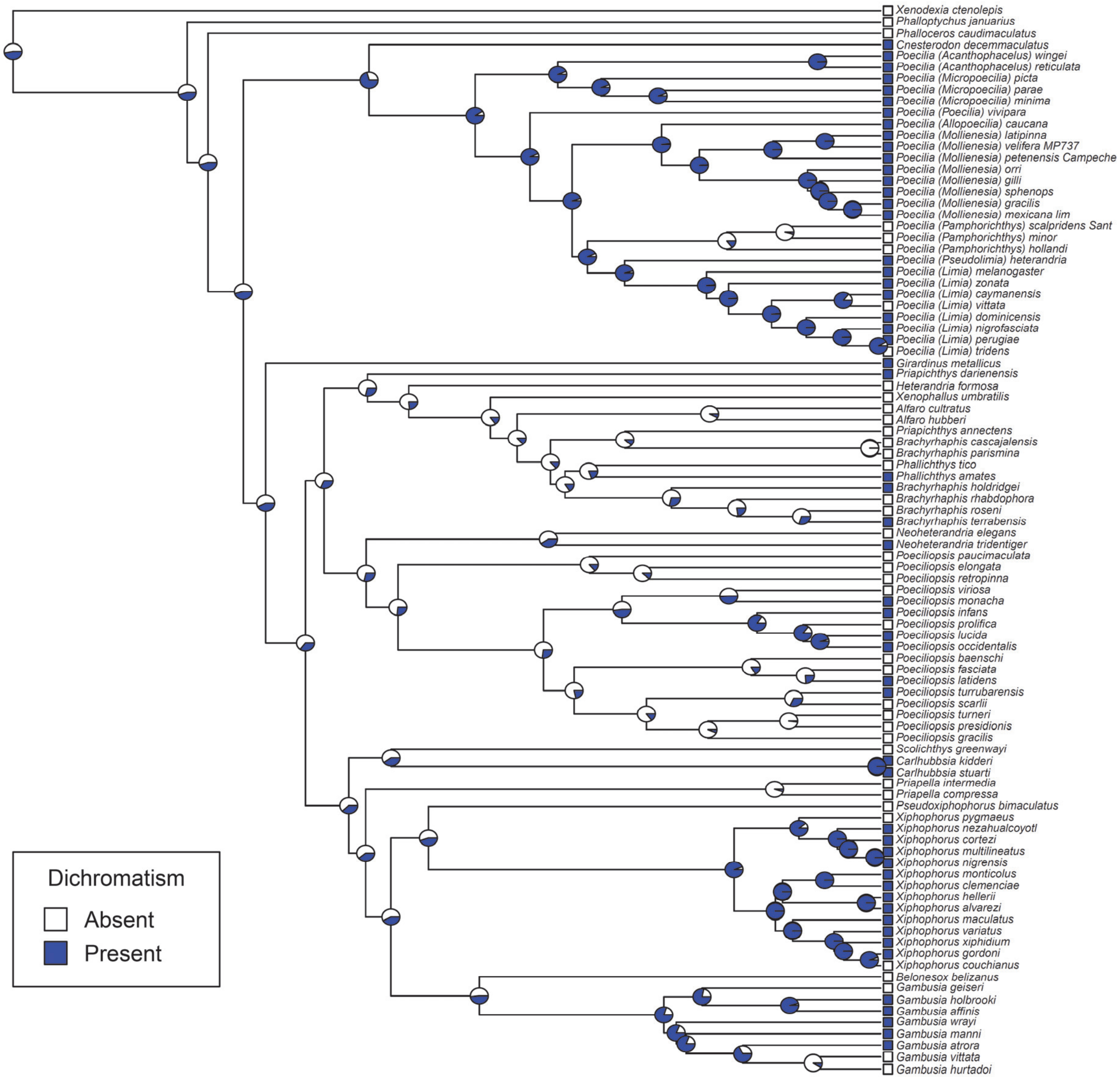

Supplementary Figure 7. Maximum likelihood ancestral state reconstruction of dichromatism (absent / present) under equal rates model.

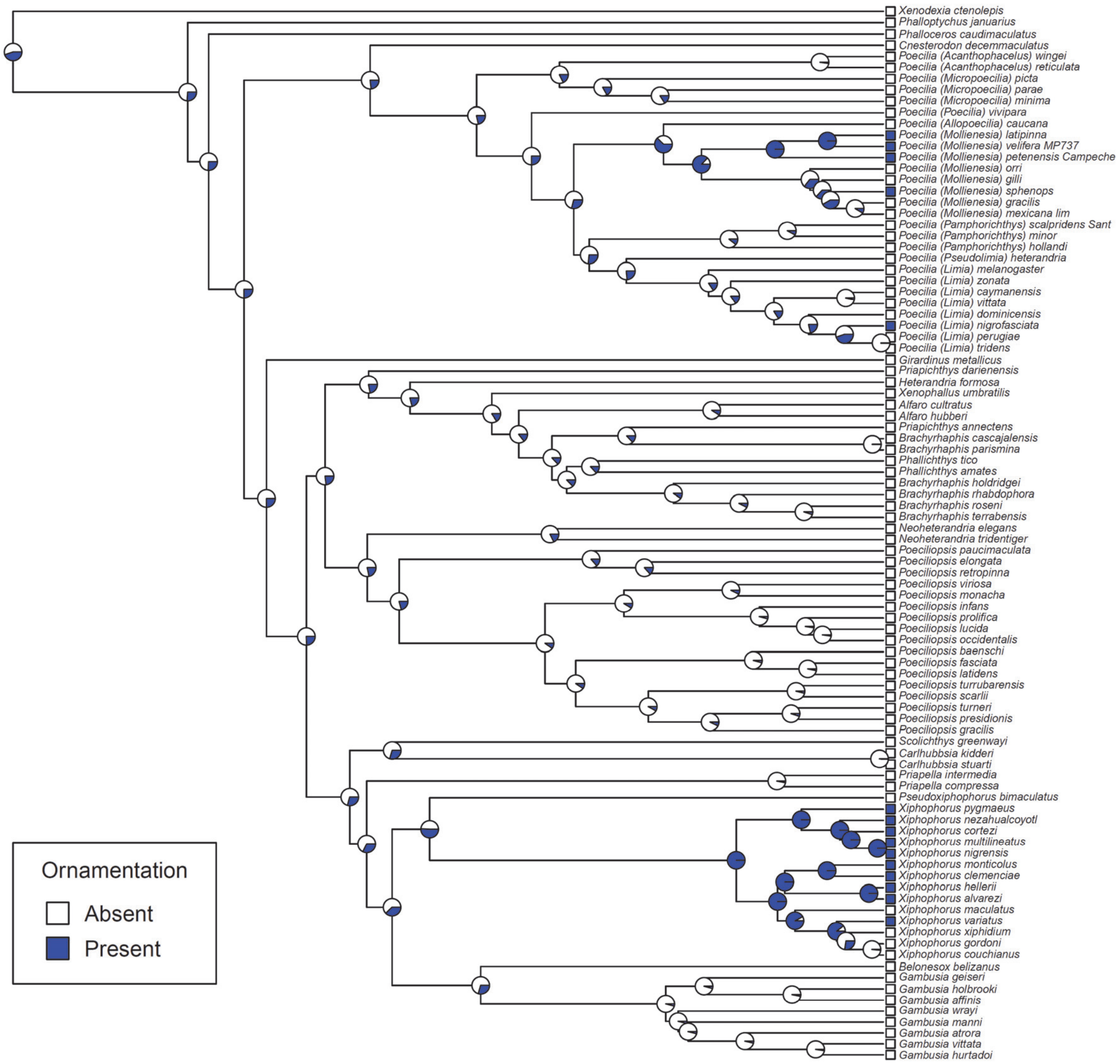

Supplementary Figure 8. Maximum likelihood ancestral state reconstruction of ornamentation (absent / present) under all rates different model.

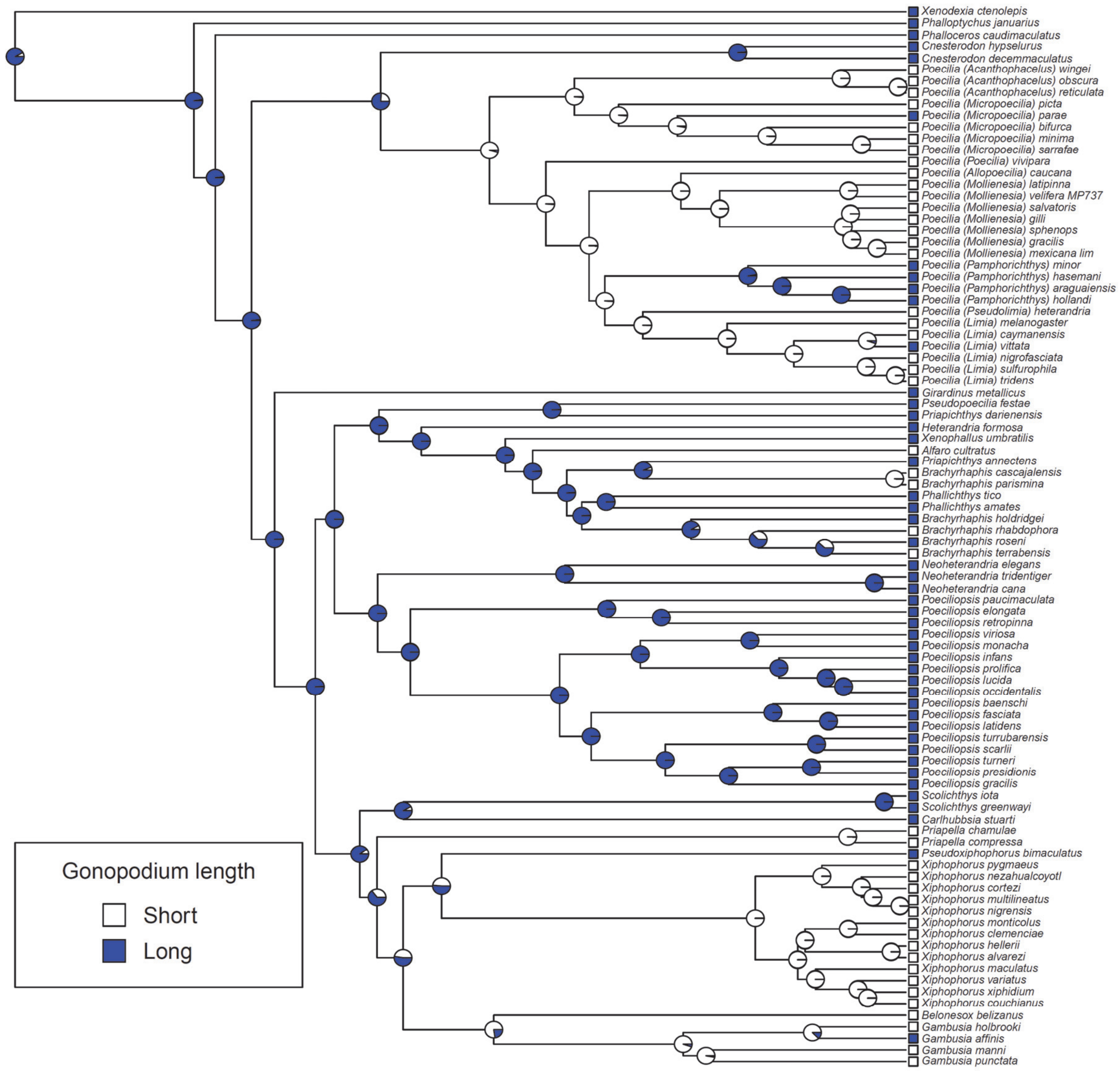

Supplementary Figure 9. Maximum likelihood ancestral state reconstruction of relative gonopodium length (short / long) under equal rates model.

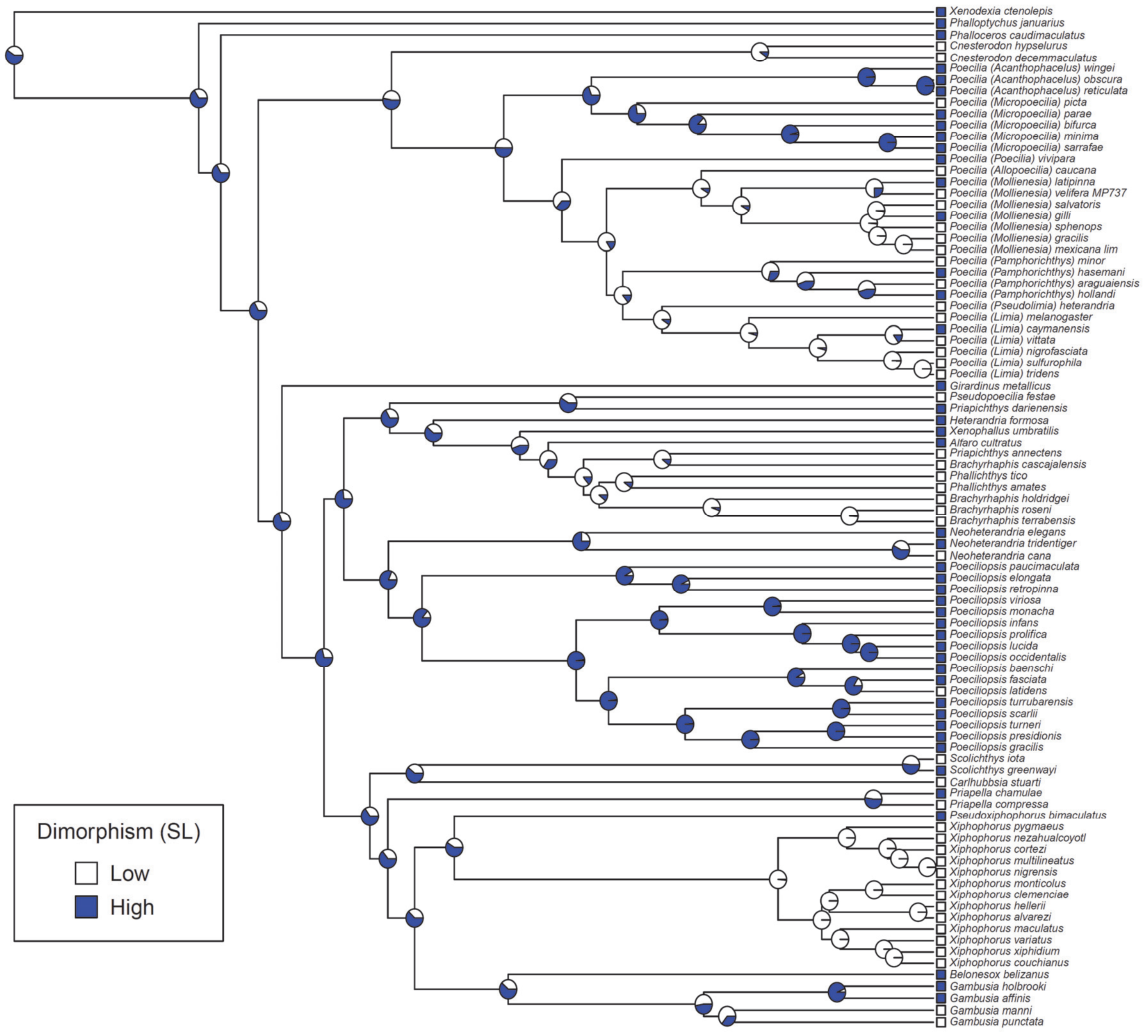

Supplementary Figure 10. Maximum likelihood ancestral state reconstruction of the sexual dimorphism index – standard length (low / high) under equal rates model.

Supplementary Note 3: Joint evolution of male and female traits

This section provides additional details regarding the joint evolution of the placenta and male traits associated with pre-copulatory mate choice. We first illustrate their joint evolution by presenting mirrored trees showing paired reconstructions of each male trait and placentation (Supplementary Figures 11-15). We then show results of BayesTraits Discrete analyses that establish the dependency between the evolution of female mode of reproduction (i.e. placentation) and some male traits (Supplementary Table 5). Finally, we present the results of two additional checks on the robustness of the BayesTraits results (Supplementary Tables 6-8 and Supplementary Figures 16-18).

First, we tested how fixing the root state influenced the results of our BayesTraits Discrete Dependent modeling of the correlated evolution of male traits and the placenta. BayesTraits analyses on the correlated evolution of male traits and placentation were necessarily limited to species in the Poeciliinae for which we had matched male and female trait data. As a consequence of excluding outgroup taxa there was uncertainty in the estimated root state of the tree. We were concerned that this uncertainty may have substantial influence on the results, given the complexity at the root of the tree (e.g., *Xenodexia* is placental, *Tomeurus* lays eggs). To test the effect the root state had on the results, we compared our initial analyses in which the root state was freely estimated from the data (and hence exhibited a lot of uncertainty), with identical analyses in which we fixed the root as the most likely state – no courtship and no placenta, no dichromatism and no placenta, no ornamentation and no placenta, long gonopodium and no placenta, high sexual dimorphism and no placenta – see Supplementary Tables 2 and 4. Fixing the root state did not influence support for correlated evolution in comparisons between BayesTraits Discrete Dependent and Independent models (Supplementary Table S6), and transition rate estimates derived from BayesTraits Discrete Dependent models were similar with unfixed and fixed root state (Supplementary Figure S16).

Secondly, we performed a sensitiviety analysis to determine how the choice of criterion used to define long and short gonopodium length (PROPGL), and high and low sexual dimorphism standard length (SDISL), influenced BayesTraits Discrete models. The continuous traits of gonopodium length and sexual dimorphism index standard length were converted to binary characters (0/1) by scoring the bottom 50% of species as small (i.e. state 0) and the top 50% as large (i.e. state 1). We feel this is justifiable because there were no distinct breaks in the distribution of these traits (Supplementary Figure 17). Nonetheless, we were concerned that the choice of cutoff could be driving these results. We therefore performed a sensitivity analysis in which we applied additional cutoff criteria to each of these traits and repeated the BayesTraits Discrete analyses. Evidence for correlated evolution between binary characters (i.e. support for Dependent model) was, in general, unaffected by the choice of cut-off criteria (Supplementary Table 7), and the best-supported evolutionary pathways derived from BayesTraits Discrete Dependent analysis of SDISL and the placenta were the same as those with the original 50/50 cut-off criteria (Supplementary Table 8, Supplementary Figure 18).

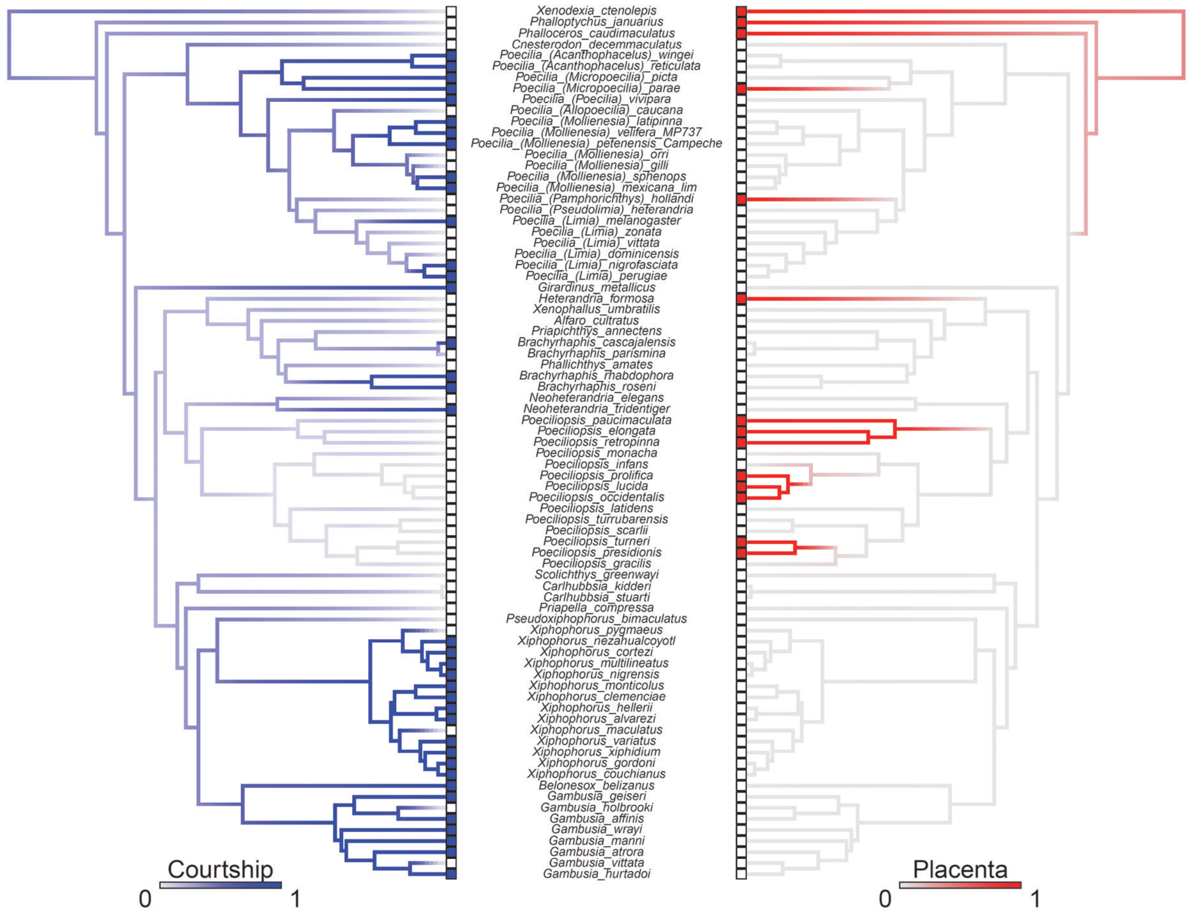

Supplementary Figure 11. Mirrored trees illustrating correlated evolution between courtship and the placenta. Ancestral state reconstructions of courtship and the placenta by stochastic character mapping. Phylogeny restricted to species with both courtship and placentation data (n=79). Branch colors represent posterior probability densities of edge states based on 1000 stochastic character maps of each reconstruction.

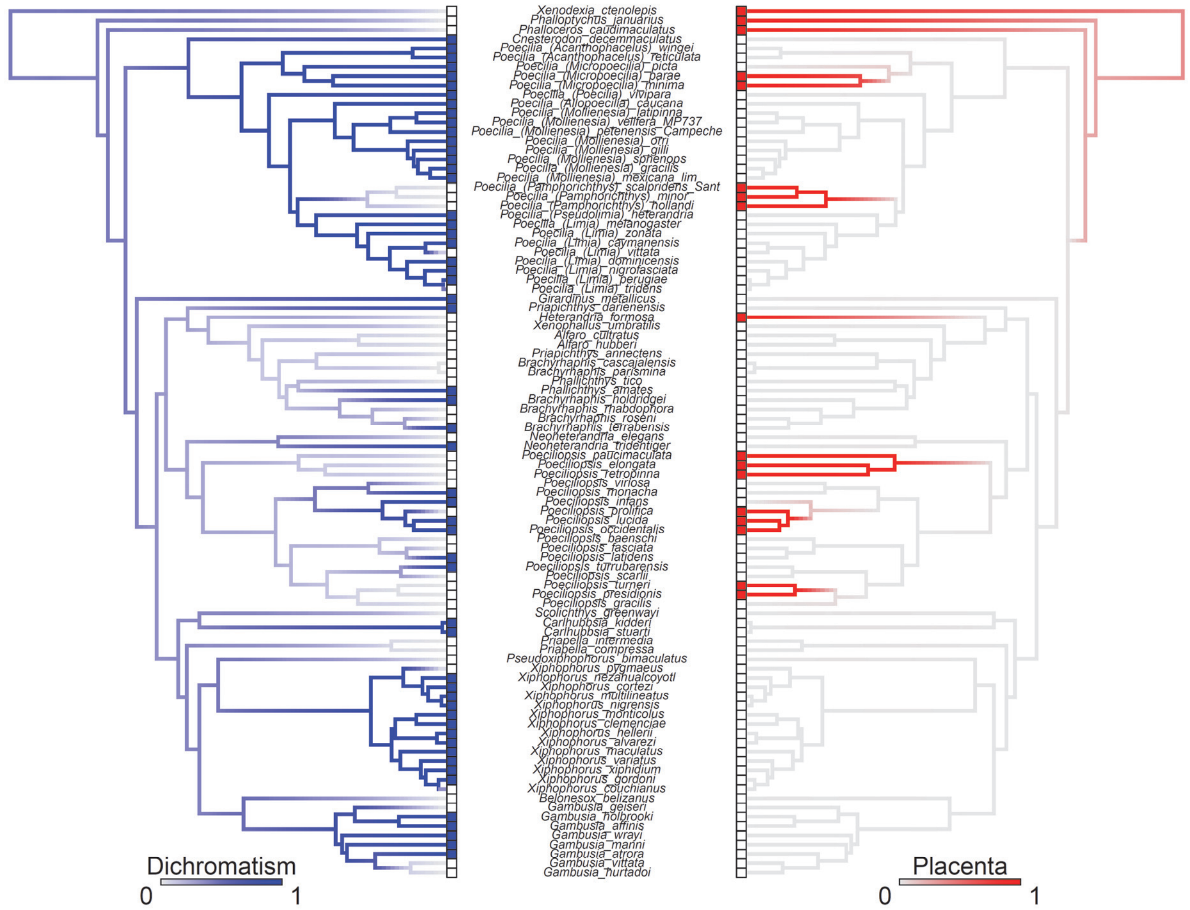

Supplementary Figure 12. Mirrored trees illustrating correlated evolution between dichromatism and the placenta. Ancestral state reconstructions of dichromatism and the placenta by stochastic character mapping. Phylogeny restricted to species with both dichromatism and placentation data (n=94). Branch colors represent posterior probability densities of edge states based on 1000 stochastic character maps of each reconstruction.

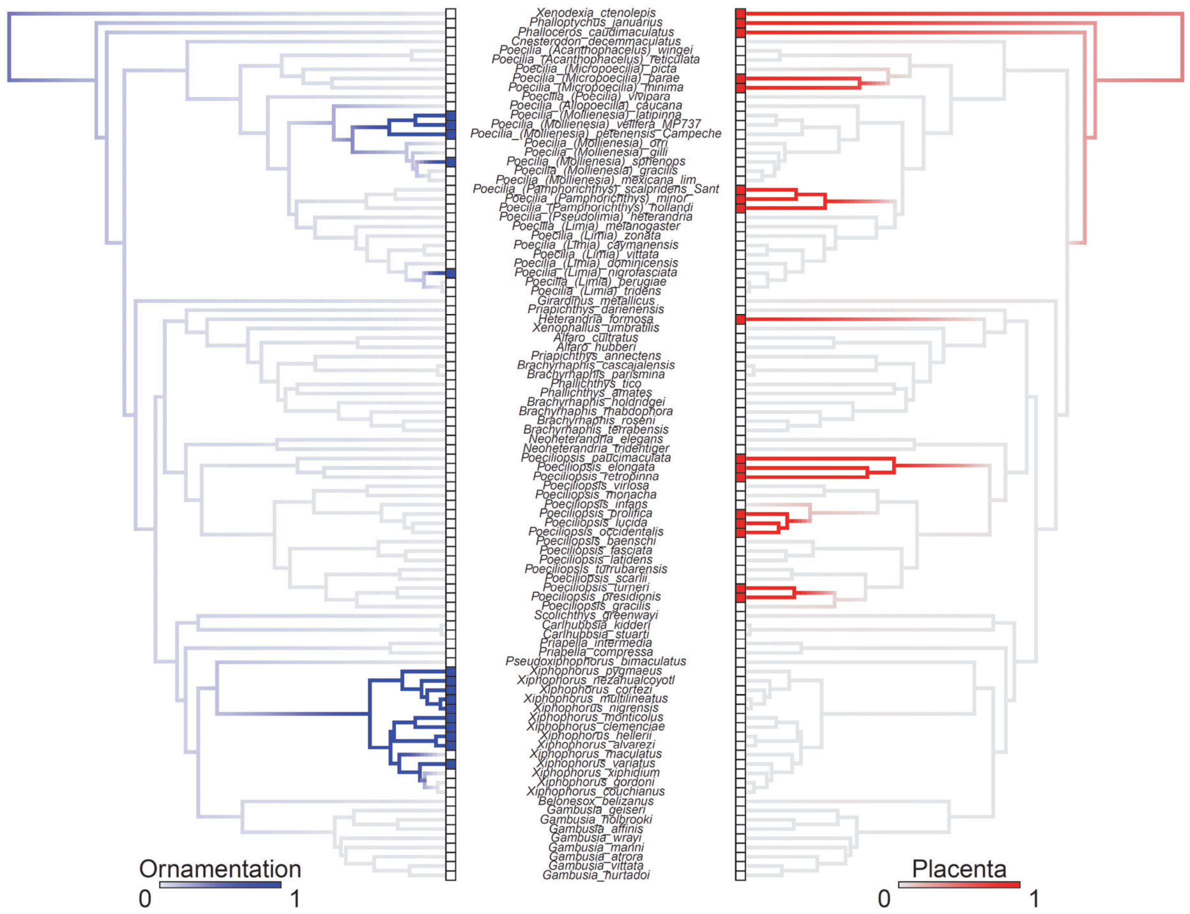

Supplementary Figure 13. Mirrored trees illustrating correlated evolution between ornamentation and the placenta. Ancestral state reconstructions of ornamentation and the placenta by stochastic character mapping. Phylogeny restricted to species with both ornamentation and placentation data (n=94). Branch colors represent posterior probability densities of edge states based on 1000 stochastic character maps of each reconstruction.

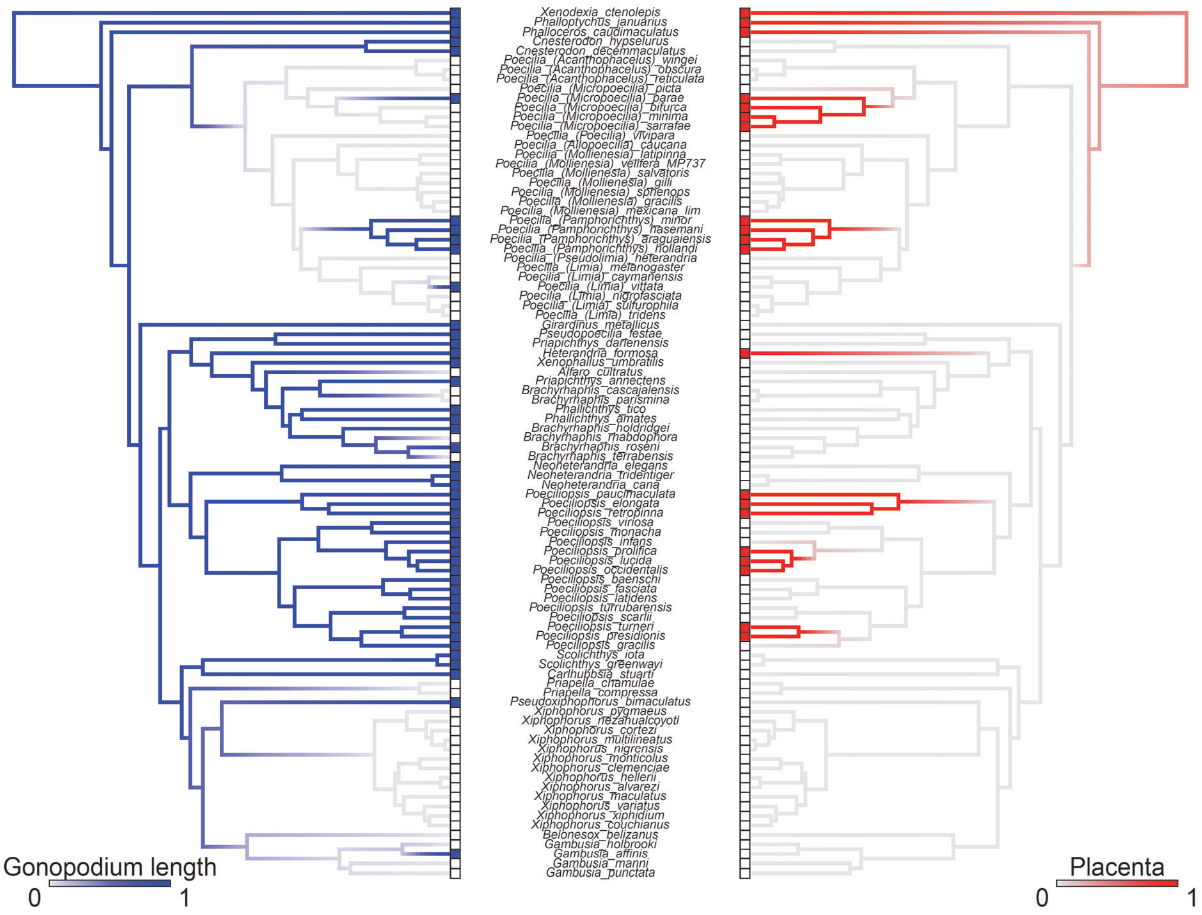

Supplementary Figure 14. Mirrored trees illustrating correlated evolution between relative gonopodium length and the placenta. Ancestral state reconstructions of gonopodium length and the placenta by stochastic character mapping. Phylogeny restricted to species with both gonopodium length and placentation data (n=92). Branch colors represent posterior probability densities of edge states based on 1000 stochastic character maps of each reconstruction.

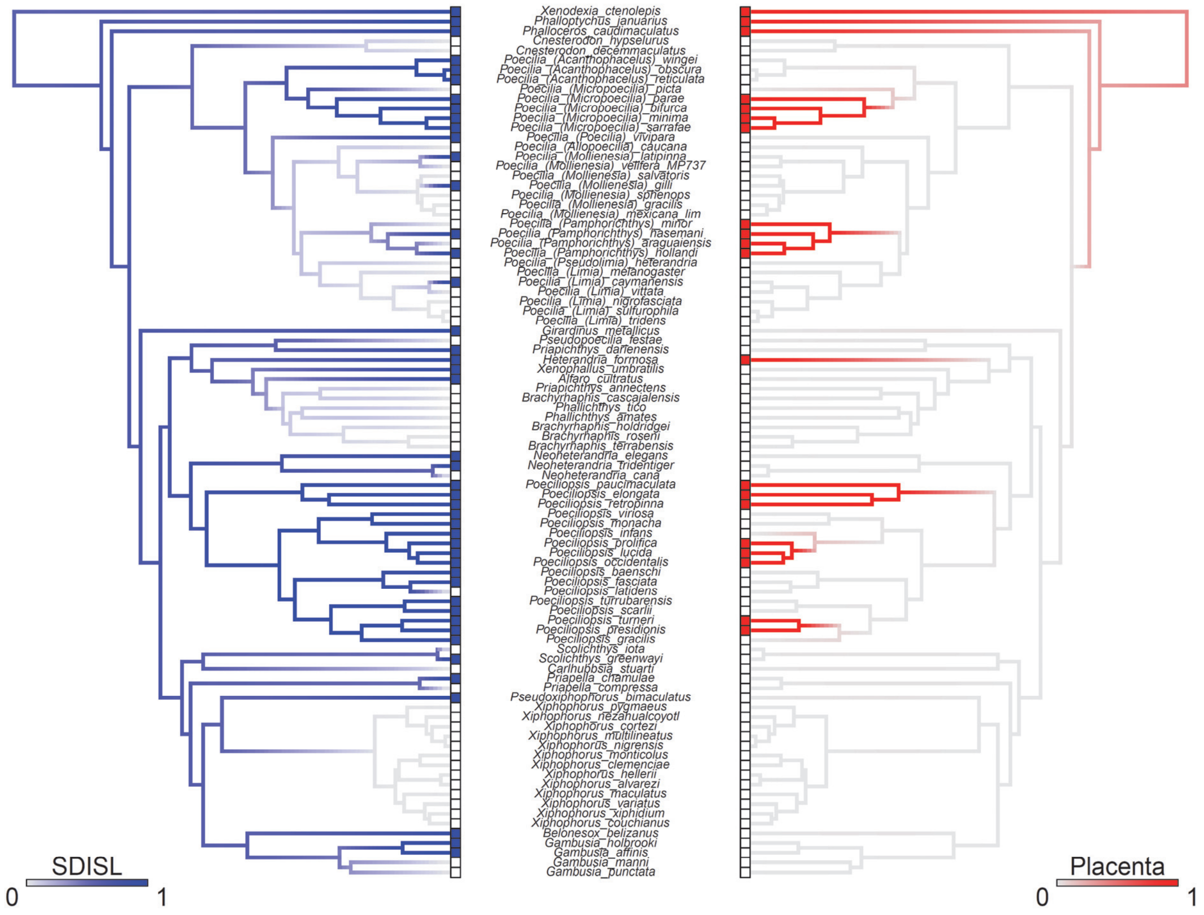

Supplementary Figure 15. Mirrored trees illustrating correlated evolution between sexual dimorphism index (standard length) and the placenta. Ancestral state reconstructions of dimorphism and the placenta by stochastic character mapping. Phylogeny restricted to species with both dimorphism and placentation data (n=90). Branch colors represent posterior probability densities of edge states based on 1000 stochastic character maps of each reconstruction.

Character 1	Character 2	Dependent	Independent	BF
Courtship	Placenta	-79.383	-81.479	**4.192**
Dichromatism	Placenta	-95.864	-97.706	**3.682**
Ornamentation	Placenta	-62.144	-62.222	0.156
Gonopodium length	Placenta	-71.571	-70.928	-1.286
SDISL	Placenta	-85.818	-89.988	**8.340**

Supplementary Table 5. Comparison of BayesTraits Discrete Dependent (8-parameter) and Independent (4-parameter) models of male traits & placentation using a stepping stone sampler. The log marginal likelihood of the Dependent and Independent models was compared using log Bayes factors. Bayes factors greater than 2 are considered positive evidence for the model with the higher harmonic mean, greater than 5 as strong evidence, while values over 10 as very strong evidence. Evidence for correlated evolution between binary characters (i.e. support for Dependent model) was found for courtship & placentation, dichromatism & placentation, and sexual dimorphism index standard length (SDISL) & placentation.

(a) Unfixed root				
**Character 1**	**Character 2**	**Dependent**	**Independent**	**BF**
Courtship	Placenta	-79.383	-81.479	**4.192**
Dichromatism	Placenta	-95.864	-97.706	**3.682**
Ornamentation	Placenta	-62.144	-62.222	0.156
Gonopodium length	Placenta	-71.571	-70.928	-1.286
SDISL	Placenta	-85.818	-89.988	**8.340**

(b) Fixed root				
**Character 1**	**Character 2**	**Dependent**	**Independent**	**BF**
Courtship	Placenta	-80.699	-82.127	**2.857**
Dichromatism	Placenta	-97.181	-98.634	**2.907**
Ornamentation	Placenta	-62.498	-62.942	0.888
Gonopodium length	Placenta	-71.914	-71.473	-0.883
SDISL	Placenta	-88.755	-92.685	**7.860**

Supplementary Table 6. Comparison of BayesTraits Discrete Dependent (8-parameter) and Independent (4-parameter) models of sexually selected traits & placentation using a stepping stone sampler. The log marginal likelihood of the Dependent and Independent models was compared using log Bayes factors. Bayes factors greater than 2 are considered positive evidence for the model with the higher harmonic mean, greater than 5 as strong evidence, while values over 10 as very strong evidence. Evidence for correlated evolution between binary characters (i.e. support for Dependent model) was found for courtship & placentation, dichromatism & placentation, and sexual dimorphism & placentation. Fixing the root state did not influence the results.

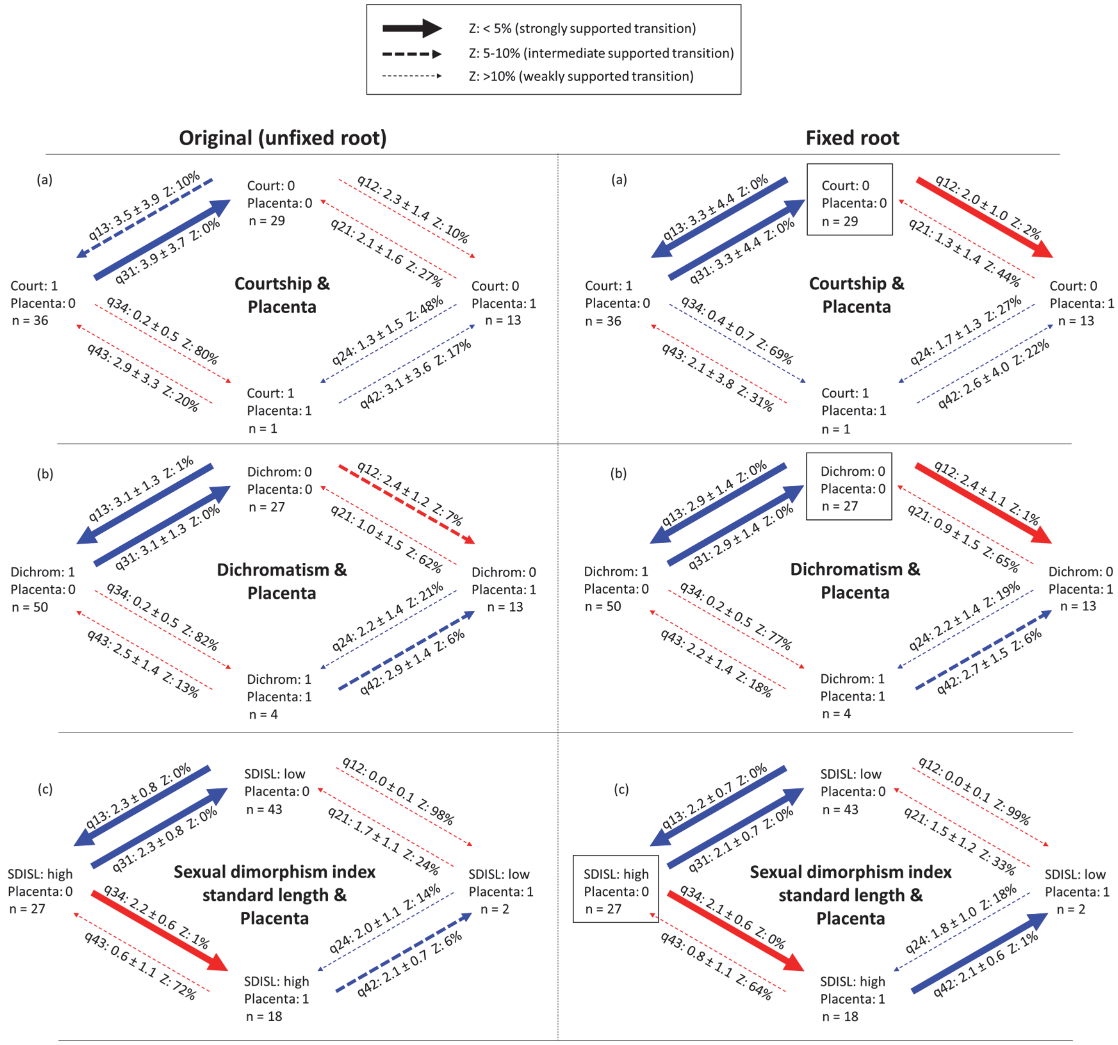

Supplementary Figure 16. Summary of transition rate estimates from BayesTraits Discrete Dependent models of character evolution examining the joint evolution of the placenta and (a) courtship, (b) dichromatism, (c) sexual dimorphism index standard length. Mean values, standard deviations, and the percentage of models in which each transition rate had a value of zero (Z) are summarized from the posterior distribution. Solid arrows indicate the best-supported evolutionary pathways in which Z-scores are less than 5%. Dashed arrows indicate less well-supported pathways. Blue arrows indicate a change in male traits while female traits remain the same; conversely, red arrows indicate a change in female traits while male traits remain the same. Here, a side-by-side comparison can be seen of the effect fixing the root state had on estimated transition rates.

BayesTraits Discrete Dependent model results were similar with unfixed and fixed root state. For analyses on courtship and placentation, and dichromatism and placentation, fixing the root state as non-sexually selected and non-placental resulted in increased support for the transition q12 (placenta evolving in lineages lacking sexually selected traits), with the other transition rates remaining largely unchanged. This pathway is likely to have been strengthened because BayesTraits may now be inferring independent origins of the placenta in each of the basal placental lineages that lack sexually selected traits. With the root state unfixed this pathway still received intermediate support, but there was undoubtedly more uncertainty regarding the pattern of transitions in these basal lineages, so the signal probably came predominately from the gain of placenta in non-sexually selected lineages further down the tree.

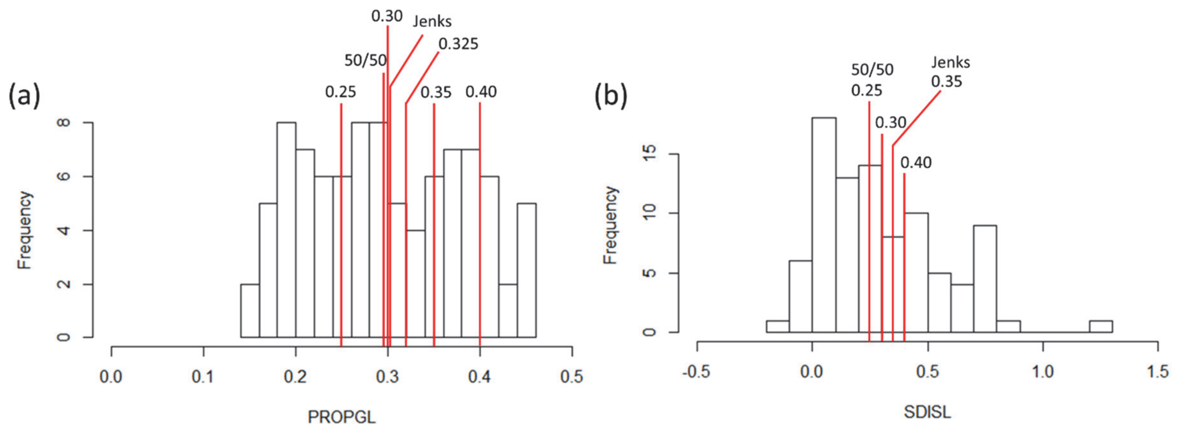

Supplementary Figure 17. Frequency plots of (a) Proportional gonopodium length (PROPGL), and (b) Sexual dimorphism index standard length (SDISL). The alternative cutoff criteria used to convert these traits to binary (0/1) characters are indicated as red lines.

(a) PROPGL and Placenta							
**Character 1**	**Character 2**	**Character 1 criterion**	**State 0**	**State 1**	**Dependent**	**Independent**	**BF**
PROPGL	Placenta	50/50 (>=0.296375)	46	46	-71.571	-70.928	-1.286
PROPGL	Placenta	Jenks (>0.3066)	52	40	-71.512	-70.928	-1.169
PROPGL	Placenta	>0.25	32	60	-63.402	-62.662	-1.480
PROPGL	Placenta	>0.30	50	42	-74.475	-73.530	-1.890
PROPGL	Placenta	>0.325	56	36	-67.450	-66.757	-1.386
PROPGL	Placenta	>0.35	61	31	-70.208	-71.219	**2.021**
PROPGL	Placenta	>0.40	79	13	-64.471	-64.043	-0.855

(b) SDISL and Placenta							
**Character 1**	**Character 2**	**Character 1 criterion**	**State 0**	**State 1**	**Dependent**	**Independent**	**BF**
SDISL	Placenta	50/50 (>=0.24855)	45	45	-85.818	-89.988	**8.340**
SDISL	Placenta	Jenks (>0.3483)	54	36	-87.854	-92.511	**9.313**
SDISL	Placenta	>0.25	45	45	-85.833	-89.988	**8.310**
SDISL	Placenta	>0.30	52	38	-87.181	-92.104	**9.845**
SDISL	Placenta	>0.35	54	36	-87.841	-92.502	**9.323**
SDISL	Placenta	>0.40	60	30	-87.789	-92.865	**10.154**

Supplementary Table 7. Test of correlated evolution and sensitiviety analysis for the criterion used to define gonopodium length (PROPGL), and sexual dimorphism index standard length (SDISL). Character 1 criterion shows the cutoff points used for each trait – the original 50/50 cirtierion and five or six others specific to the given trait. State 0 and 1 indicates the number of species in each state for the given criterion. The log marginal likelihood of the Dependent (8-parameter) and Independent (4-parameter) models were compared using log Bayes factors. Bayes factors greater than 2 are considered positive evidence for the model with the higher harmonic mean, greater than 5 as strong evidence, while values over 10 as very strong evidence. Evidence for correlated evolution between binary characters (i.e. support for Dependent model) was not found for gonopodium length and the placenta, with the exception of a single cutoff criteria (>0.35). Evidence for correlated evolution between binary characters (i.e. support for Dependent model) was found for sexual dimorphism (SDISL) and the placenta irrespective of the cutoff criteria utilized.

SDISL and Placenta					
**Character 1**	**Character 2**	**Character 1 criterion**	**Strongly supported transitions [% Zero: < 5%]**	**Intermediate supported transitions [% Zero: 5-10%]**	**Weakly supported transitions [% Zero: >10%]**
SDISL	Placenta	50/50	q13,q31,q34	q42	q12,q21,q24,q43
SDISL	Placenta	Jenks	q13,q31,q34,q42	.	q12,q21,q24,q43
SDISL	Placenta	>0.25	q13,q31,q34	q42	q12,q21,q24,q43
SDISL	Placenta	>0.30	q13,q31,q34,q42	.	q12,q21,q24,q43
SDISL	Placenta	>0.35	q13,q31,q34,q42	.	q12,q21,q24,q43
SDISL	Placenta	>0.40	q13,q31,q34,q42	.	q12,q21,q24,q43

Supplementary Table 8. Supported and unsupported transition rate estimates derived from BayesTraits Discrete Dependent analysis of sexual dimorphism index standard length (SDISL) & placenta using different cutoff criteria.

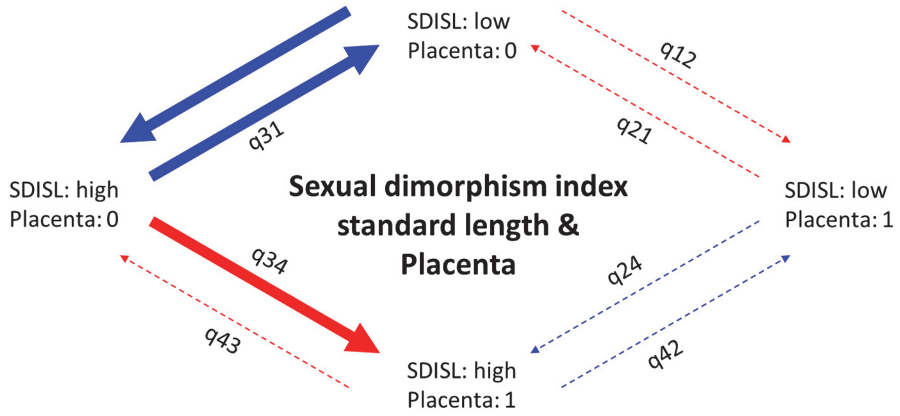

Supplementary Figure 18. Summary of best-supported transition rate estimates [% Zero < 5%] from BayesTraits Discrete Dependent models of character evolution examining the joint evolution of sexual dimorphism index standard length (SDISL) and the placenta. Bold arrows indicate the best-supported evolutionary pathways, in which Z-scores are less than 5%, supported by ≥ 5 cutoff criteria (Supplementary Table 8). Here we consider transitions that are supported across multiple cut-off criteria as those likely to be most robust. Sexual dimorphism (low/high) is gained and lost in non-placental lineages (q13 & q31), and the placenta is gained in high dimorphism lineages (q34).

Supplementary Note 4: Speciation rate

This section provides additional details for analyses of the effects of male (courtship, dichromatism, ornamentation, and sexual selection index) and female (placentation) traits on the rate of speciation. First, we present maximum likelihood model comparisons of state-dependent speciation and extinction models utilizing two different methods of accounting for incomplete taxon sampling (Table S9). Next, we present Bayesian parameter estimates from binary state speciation and extinction models that utilize the unresolved tip method to account for incomplete taxon sampling (Supplementary Figure 19); these complement the estimates provided in the main text (Figure 5) which utilize the sampling fraction method of accounting for incomplete taxon sampling. Diversification analyses can be prone to type 1 error, so we present two additional analyses (Supplementary Tables 10 and 11) that establish the robustness of the results. We then present a formal cross tabulation of the male sexual selection index versus female mode of reproduction (Supplementary Table 12). Lastly, we present a maximum likelihood analysis on the joint contribution of male and female traits to the rate of speciation that indicates male sexually selected traits are associated with a higher speciation rate and female placentation is not (Supplementary Table 13).

	UNRESOLVED TIPS						SAMPLING FRACTION					
**PLACENTOTROPHY (BiSSE)**												
**Model**	**Df**	**lnLik**	**AIC**	**ChiSq**	**Pr(>|Chi|)**	**Significance**	**Df**	**lnLik**	**AIC**	**ChiSq**	**Pr(>|Chi|)**	**Significance**
Full	6	-78.552	169.100				6	124.600	-237.200			
Equal speciation	5	-80.087	170.170	3.070	0.07974	.	5	122.240	-234.480	4.720	0.02981	*
Equal diversification	4	-80.443	168.890	3.783	0.15082		4	121.690	-235.370	5.827	0.05429	.
**COURTSHIP (BiSSE)**												
**Model**	**Df**	**lnLik**	**AIC**	**ChiSq**	**Pr(>|Chi|)**	**Significance**	**Df**	**lnLik**	**AIC**	**ChiSq**	**Pr(>|Chi|)**	**Significance**
Full	6	-72.757	157.510				6	24.755	-37.511			
Equal speciation	5	-77.128	164.260	8.741	0.00311	**	5	18.988	-27.976	11.535	0.00068	***
Equal diversification	4	-82.986	173.970	20.457	0.00004	***	4	10.990	-13.981	27.530	0.00000	***
**DICHROMATISM (BiSSE)**												
**Model**	**Df**	**lnLik**	**AIC**	**ChiSq**	**Pr(>|Chi|)**	**Significance**	**Df**	**lnLik**	**AIC**	**ChiSq**	**Pr(>|Chi|)**	**Significance**
Full	6	-74.521	161.040				6	27.222	-42.443			
Equal speciation	5	-84.521	179.040	20.000	0.00001	***	5	21.942	-33.884	10.559	0.00116	**
Equal diversification	4	-86.193	180.390	23.344	0.00001	***	4	15.829	-23.658	22.785	0.00001	***
**ORNAMENTATION (BiSSE)**												
**Model**	**Df**	**lnLik**	**AIC**	**ChiSq**	**Pr(>|Chi|)**	**Significance**	**Df**	**lnLik**	**AIC**	**ChiSq**	**Pr(>|Chi|)**	**Significance**
Full	6	-55.566	123.130				6	59.470	-106.940			
Equal speciation	5	-68.671	147.340	26.210	0.00000	***	5	56.722	-103.445	5.495	0.01907	*
Equal diversification	4	-69.036	146.070	26.941	0.00000	***	4	50.841	-93.681	17.259	0.00018	***
**SEXUAL SELECTION INDEX (MuSSE)**												
**Model**	**Df**	**lnLik**	**AIC**	**ChiSq**	**Pr(>|Chi|)**	**Significance**	**Df**	**lnLik**	**AIC**	**ChiSq**	**Pr(>|Chi|)**	**Significance**
Full	NA	NA	NA	NA	NA	NA	20	-8.091	56.181			
Equal speciation	NA	NA	NA	NA	NA	NA	17	-20.656	75.312	25.131	0.00001	***
Equal diversification	NA	NA	NA	NA	NA	NA	14	-32.533	93.066	48.885	0.00000	***

Significance codes: 0 '***' 0.001 '**' 0.01 '*' 0.05 '.' 0.1 ' ' 1

Supplementary Table 9. Comparison of full and constrained maximum-likelihood binary-state speciation and extinction (BiSSE) and multi-state speciation and extinction (MuSSE) models. The full model includes trait-dependent diversification (i.e. separate speciation rates and extinction rates estimated for trait presence and absence, as well as 2 transition rate parameters for gain and loss of the trait). The equal speciation rate model is constrained such that a single speciation rate is estimated for presence and absence of the trait. The equal diversification model is constrained such that a single speciation rate and single extinction rate is estimated for presence and absence of the trait. Full and constrained models are compared using AIC and Chi-square test of the log-likelihoods. Support for the full model indicates trait-dependent speciation and/or diversification rates. Two methods were utilized to account for incomplete taxon sampling – unresolved tips and sampling fraction (see methods). Both methods yield similar results. In addition to maximum likelihood, BiSSE and MuSSE analyses were also conducted in a Bayesian mcmc framework. Supplementary Figure 19 displays the mcmc posterior distributions of all parameter estimates derived from the full models, utilizing the unresolved tips method of accounting for incomplete taxon sampling; Figure 5, in the main text, presents this information while utilizing the sampling fraction method of accounting for incomplete taxon sampling.

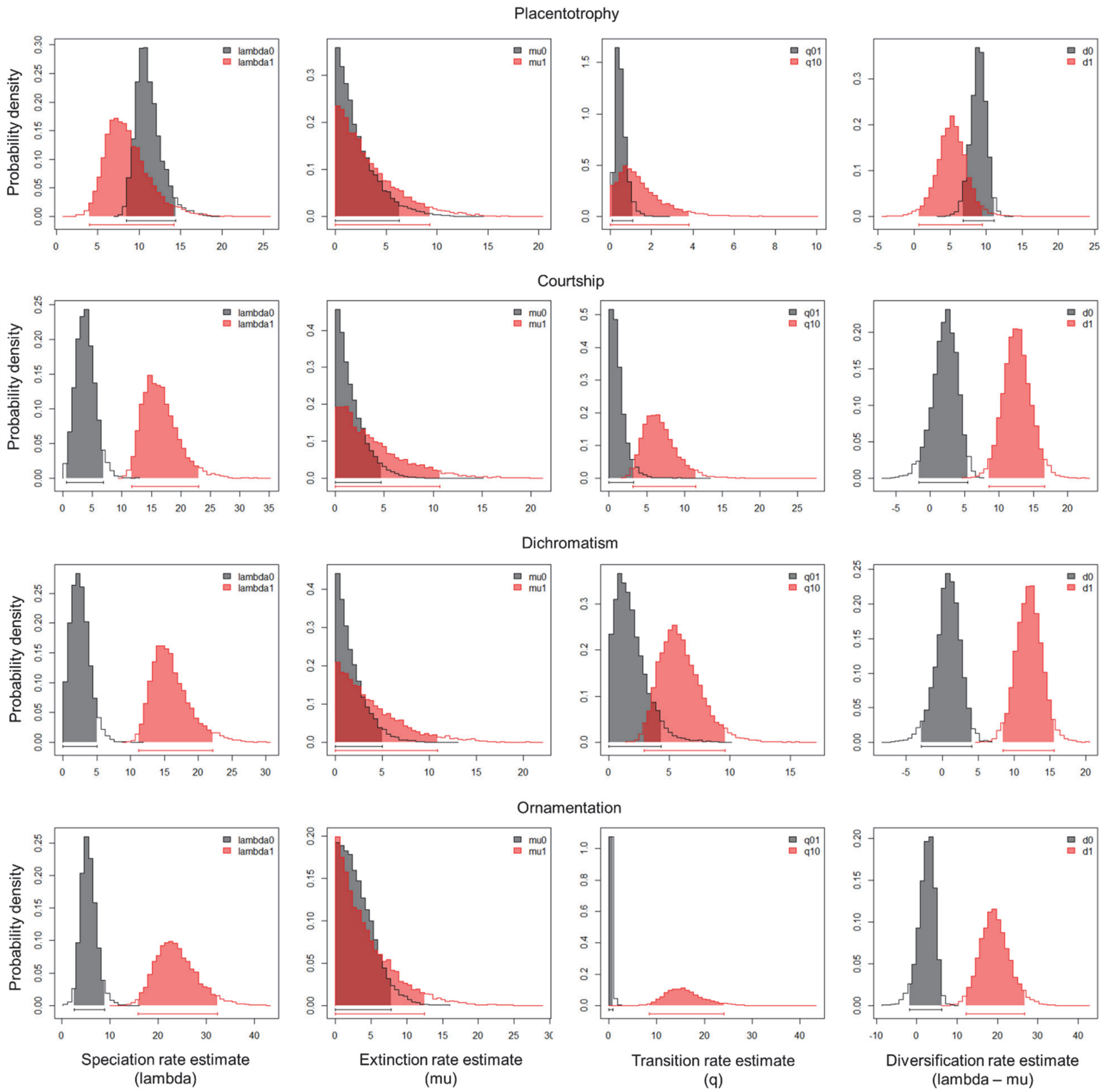

Supplementary Figure 19. Bayesian parameter estimates inferred using the six-parameter BiSSE model for each of four binary traits - placentotrophy, courtship, dichromatism, and ornamentation. The unresolved tips method was used to account for incomplete taxon sampling (see methods). Zero (0) indicates absence of trait and one (1) indicates presence. Estimates of: trait-specific speciation rates (lambda); trait-specific extinction rates (mu); transition rates (q); net diversification rates calculated as the difference between speciation (lambda) and extinction (mu) rates. The 95% credibility intervals are indicated as horizontal colored bars above the x-axis. The presence of courtship, dichromatism, and ornamentation are each associated with higher rates of speciation and diversification.

Trait	lambda0	lambda1	null_mean_diff	null_sd	nchanges_parsimony	qpars	pval_2tailed
Placentotrophy	15.902	8.124	-0.406	7.385	8	0.532	0.37962
Courtship	6.642	13.907	-0.030	3.540	16	3.164	0.00400
Dichromatism	8.185	12.652	-0.001	3.209	22	3.397	0.18581
Ornamentation	9.355	18.106	-0.137	5.078	6	0.455	0.03596

Supplementary Table 10. Results of FiSSE: A simple nonparametric test for the effects of a binary character on lineage diversification rates[3]. Lambda0 and lambda1 are “quasi-parameters” that are correlated with, but not identical to, underlying rates of speciation. Using this conservative method, courtship and ornamentation are found to be correlated with significantly higher rates of speciation (pval_2tailed). Dichromatism and a lack of placentotrophy are also associated with higher speciation, but are not significant.

Trait	Number of significant results ($p < 0.05$) in simulation comparisons	Observed diversitree *p*-value	Probability of significant effect of diversification rate
Placentotrophy	27 / 100	0.05429000	$\leq$ 0.29
Courtship	57 / 100	0.00000105	$\leq$ 0.04
Dichromatism	53 / 100	0.00001128	$\leq$ 0.02
Ornamentation	16 / 100	0.00017880	$\leq$ 0.01

Supplementary Table 11. Comparison of simulation output designed to test the level of type 1 error in BiSSE trait-dependent diversification analyses. Given the real tree and a hypothetical trait with two states and transition probabilities between the two states derived from the real data, we simulated the evolution of each trait 100 times, each time beginning with the predicted ancestral state, which is the absence of the trait. We compared two models, one in which the speciation and extinction rates of each character state are forced to be equal and one in which they are allowed to be different from one another. For each simulated outcome, we evaluated the data for the fit of the two models, assigned the probability that the unequal rates model was a better fit, then generated a frequency distribution for these probabilities. We expect that there should be a significant difference only 5% of the time. We actually observed apparent significance in far more than 5% of the simulated trees, so there was clear evidence of type 1 errors when applying diversitree. We then used this frequency distribution of *p*-values to determine what *p*-value corresponds to a 5% cut off. If the observed *p*-value from the empirical data is within the 5th percentile of significance (i.e. more extreme than 95 out of 100 *p*-values generated from the model comparison on the simulated output), then this is taken as evidence of significant character-dependent diversification. By this criterion, the presence of courtship, dichromatism and ornamentation are all associated with significantly higher rates of diversification. There is not a significant difference in diversification rate between species with or without placentotrophy.

Sexual selection index	Lecithotrophic species	Placentotrophic species	Total species	Placental species in this category
0	12	11	23	*Heterandria formosa, Poecilia (Pamphorichthys) hollandi, Phalloceros caudimaculatus, Phalloptychus januarius, Poeciliopsis elongata, Poeciliopsis paucimaculata, Poeciliopsis presidionis, Poeciliopsis prolifica, Poeciliopsis retropinna, Poeciliopsis turneri, Xenodexia ctenolepis*
1	24	2	26	*Poeciliopsis lucida, Poeciliopsis occidentalis*
2	16	1	17	*Poecilia (Micropoecilia) parae*
3	13	0	13	None

Supplementary Table 12. Number and identity of species exhibiting male sexually selected traits as a function of having placentotrophy.

(a) Courtship and Placenta						
**Model**	**Df**	**lnLik**	**AIC**	**ChiSq**	**Pr(>|Chi|)**	**Significance**
Full	10	-0.785	21.570			
noC (lambdaC=0)	9	-6.952	31.903	12.333	0.00045	***
noP (lambdaP=0)	9	-0.937	19.875	0.305	0.58101	

(b) Dichromatism and Placenta						
**Model**	**Df**	**lnLik**	**AIC**	**ChiSq**	**Pr(>|Chi|)**	**Significance**
Full	10	-1.611	23.222			
noD (lambdaD=0)	9	-8.280	34.559	13.337	0.00026	***
noP (lambdaP=0)	9	-1.233	20.466	-0.756	1.00000	

(c) Ornamentation and Placenta						
**Model**	**Df**	**lnLik**	**AIC**	**ChiSq**	**Pr(>|Chi|)**	**Significance**
Full	10	38.074	-56.148			
noO (lambdaO=0)	9	28.014	-38.028	20.120	0.00001	***
noP (lambdaP=0)	9	30.755	-43.509	14.639	0.00013	***

Significance codes: 0 '***' 0.001 '**' 0.01 '*' 0.05 '.' 0.1 ' ' 1

Supplementary Table 13. Joint analyses on the effect of male and female traits on diversification. These maximum likelihood analyses were implemented in Diversitree[4] using the 'make.musse.multitrait' command. For each pair of traits, we first fit a full model including 'main effects' of the two traits on speciation and extinction and an independent model of character evolution (i.e. 4 transition rate parameters). We then fit two constrained models in which the main effect of each trait on speciation was deleted (i.e. fixed at 0). These model comparisons were designed to test whether male sexually selected traits or female reproductive mode is a better predictor of speciation rate.

For each pair of traits, deletion of the main effect of the male trait on speciation resulted in significantly poorer fit, while deletion of the main effect of the female trait (placentation) on speciation had no effect (courtship and dichromatism) or resulted in a poorer fit (ornamentation). In the analysis of (a) Courtship and Placenta and (b) Dichromatism and Placenta, the ML estimate of lambdaC and lambdaD (the main effects of courtship and dichromatism on speciation rate) were estimated as 25.970 and 14.904, respectively; while lambdaP (the main effects of placenta on speciation rate) were estimated as 1.002 and -3.625, respectively. In the two constrained models each of these parameters were fixed at 0 (equivalent to deleting the parameter) and the log likelihood of each model was compared to the full model. When lambdaC (25.970) or lambdaD (14.904) were fixed at 0 the model exhibited substantially poorer fit – indicating that species with courtship and dichromatism exhibit significantly higher speciation rate. In contrast, fixing lambdaP (1.002 and -3.625 respectively) at 0 resulted in no difference in model fit compared to the full model – indicating that species with placentation exhibited neutral / non-significant speciation rate. In the analysis of (c) Ornamentation and Placenta, lambdaO (main effect of ornamentation on speciation rate) was equal to 30.558, while lambdaP (main effect of placenta on speciation rate) was equal to -6.236. Unsurprisingly, the model in which lambdaO was fixed at 0 exhibited poor fit because species with ornamentation have high speciation rate (30.558). However, because lambdaP was slightly negative (-6.236), fixing this parameter at 0 resulted in a significantly poorer fit than when the parameter was freely estimated from the data –

indicating that species with placentation exhibited significantly lower speciation rate. Therefore, the interpretation of all three maximum likelihood model comparisons is similar – the male trait is associated with (significantly) higher speciation rate and placentation with neutral or (significantly) lower speciation rate. In addition to maximum likelihood, these joint analyses were conducted in a Bayesian mcmc framework. In Figure 7 (main text) we display the mcmc posterior distribution of the speciation main effects from the full models. Taken together, these results indicate that male sexually selected traits are associated with higher speciation rate.

Supplementary Note 5: Complementary analyses using the Matrotrophy Index, a continuous trait

The analyses reported in the main text treat the matrotrophy index as a binary trait, meaning that placentation is either present or absent. In this section we present ancestral state reconstructions (Supplementary Figure 20), correlated evolution analyses (Supplementary Figure 21, Supplementary Table 14), and diversification analyses (Supplementary Table 15, Supplementary Figure 22) utilizing the raw values of the matrotrophy index, meaning that we treat placentation as a continuous trait. We show that our results are qualitatively similar to those reported in the main text – suggesting they are not sensitive to whether matrotrophy is treated as a binary or continuous trait.

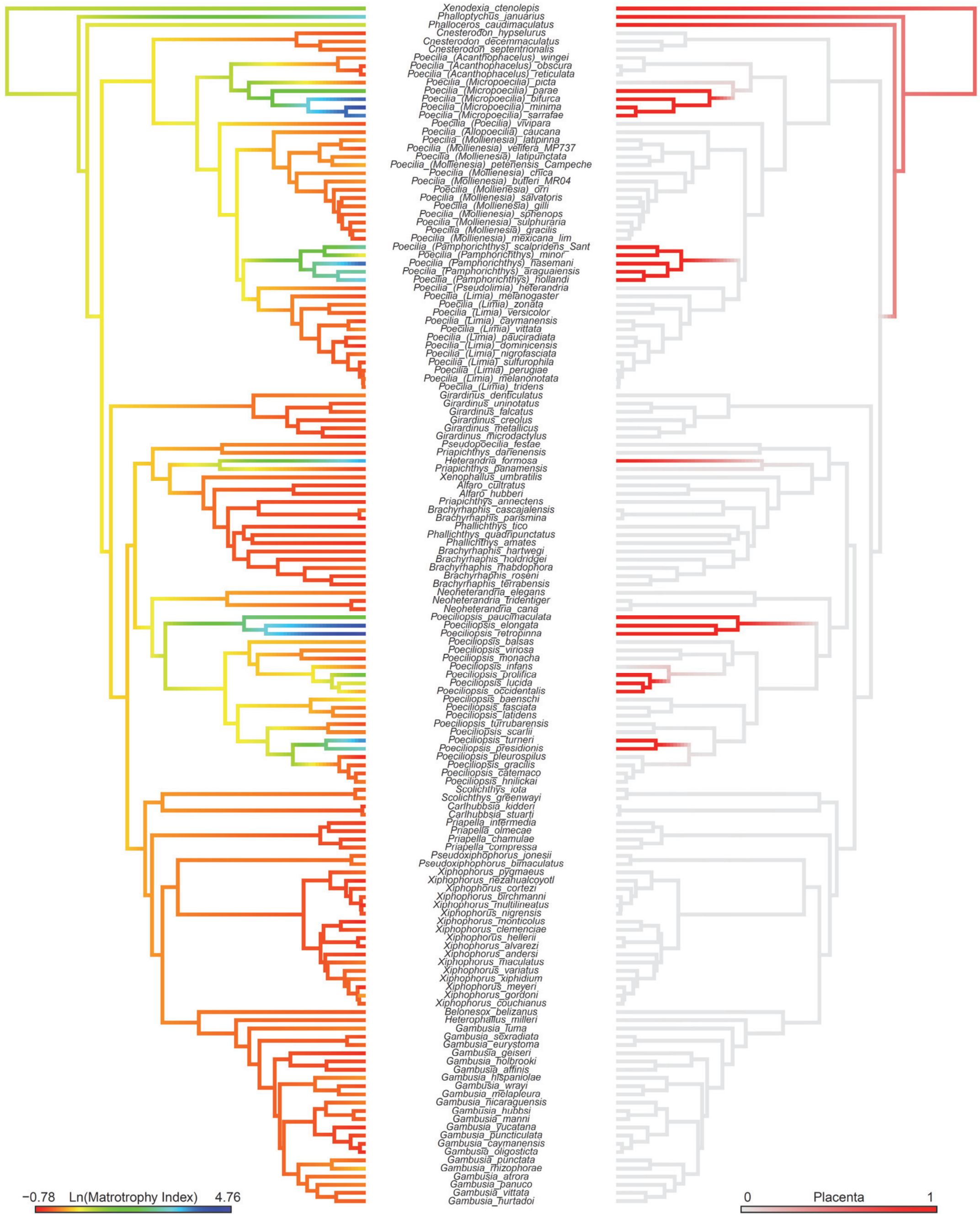

Supplementary Figure 20. Mirrored trees showing comparison between ancestral state reconstruction of the Matrotrophy Index (a continuous character) versus the placenta (a binary character). Reconstruction of Ln (Matrotrophy Index) was performed using the contMap function in the R package phytools [5], which estimates the Maximum likelihood ancestral states at all internal nodes and interpolates the states along each edge. Ancestral state reconstruction of the placenta (0/1) was performed using stochastic character mapping in the R package phytools[5]. Branch colours represent posterior probability densities of edge states based on 1000 stochastic character maps of each reconstruction.

When making interpretations regarding the root state of the subfamily and how many times placentation evolved, we feel it may be more biologically realistic to perform ancestral state

reconstructions using presence / absence of placentation rather than the continuous matrotrophy index. Firstly, the matrotrophy index is only applicable to livebearing species (i.e. it doesn't make biological sense to measure a matrotrophy index for spawning or egg-laying species). Thus, ancestral state reconstructions utilizing the matrotrophy index are limited to live-bearing taxa within the subfamily Poeciliinae (and the sister live-bearing family Anablepidae). In contrast, ancestral state reconstructions using presence / absence of placenta do not face this same limitation since non-livebearing outgroup taxa (i.e. spawning and egg-laying species) can be logically scored as non-placental. The inclusion of non-livebearing (and hence non-placental) outgroup taxa is important. As expected, given that several basal lineages in the subfamily exhibit matrotrophy, limiting the ancestral state reconstruction to Poeciliinae species gives rise to the conclusion that the root of the subfamily was most likely matrotrophic (see above). But including non-placental outgroup taxa gives rise to the conclusion that the root of the subfamily was most likely lecithotrophic (Figure 1). Secondly, the use of the continuous matrotrophy index results in the conclusion that most deeper nodes in the subfamily Poeciliinae exhibited matrotrophy (yellow coloration in above reconstruction). This implies numerous transitions from matrotrophy to lecithotrophy. We find this scenario biologically unrealistic since a transition from substantial matrotrophy to lecithotrophy necessitates the loss of the specialized embryonic and maternal tissues that define the placenta, something for which there is currently no evidence. In contrast, the use of the placenta as a binary trait results in these deeper nodes being reconstructed as non-placental, and the conclusion is that placentation has been gained numerous times in the subfamily but has not been lost (apart from some uncertainty near the root).

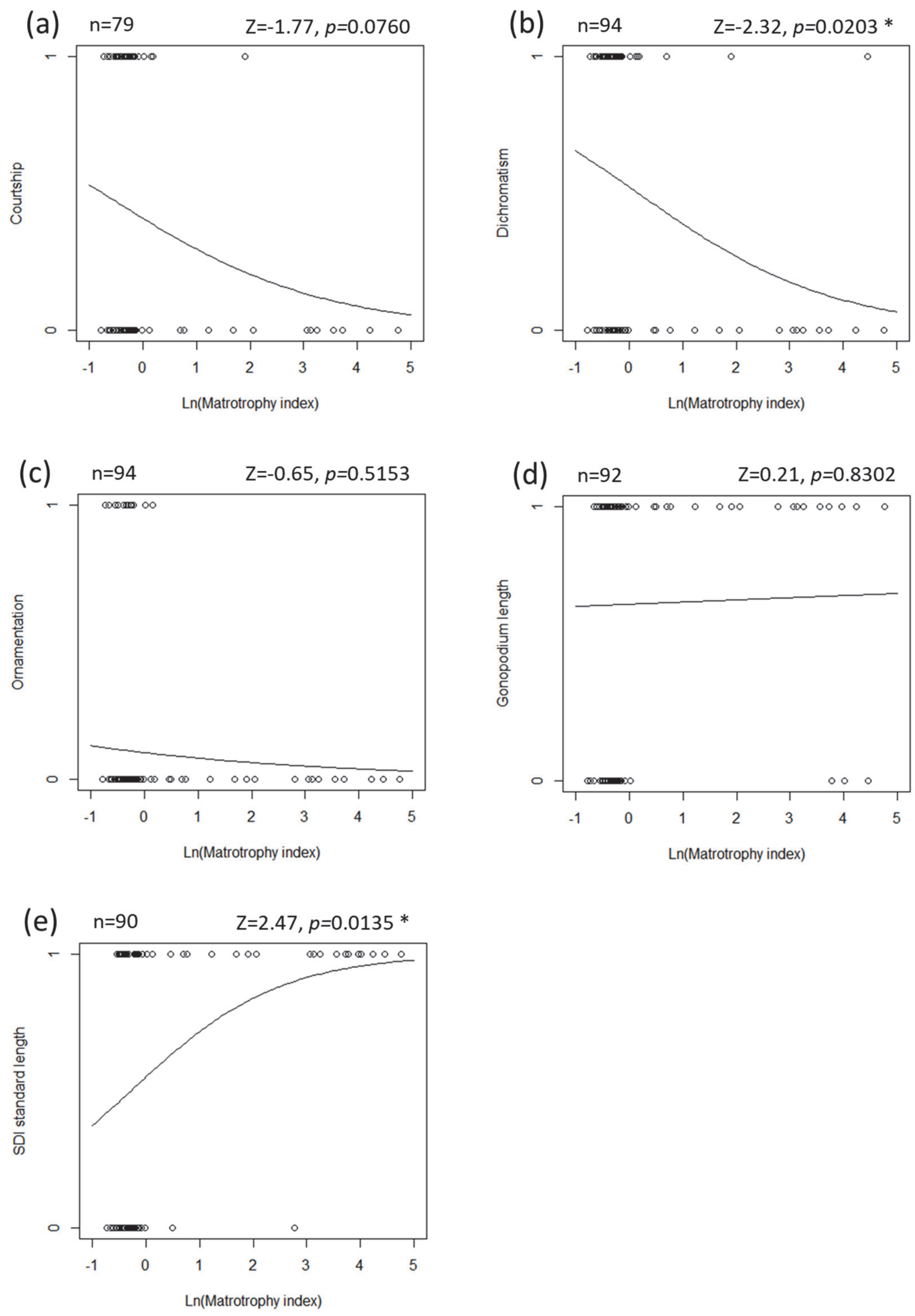

Supplementary Figure 21. Results of phylogenetic logistic regression[6] implemented in the R package phylolm[7]. In each analysis, Ln (Matrotrophy Index) is the independent variable and the male traits of (a) courtship, (b) dichromatism, (c) ornamentation, (d) relative gonopodium length, and (e) sexual dimorphism index standard length take the form of a binary response variable.

Phylogenetic logistic regression		BayesTraits Discrete	
**Variables**	**Significance (*p*<0.05)**	**Variables**	**Significance (BF>2)**
Courtship (0/1) ~ MI	N	Courtship (0/1) ~ Placenta (0/1)	Y
Dichromatism (0/1) ~ MI	Y	Dichromatism (0/1) ~ Placenta (0/1)	Y
Ornamentation (0/1) ~ MI	N	Ornamentation (0/1) ~ Placenta (0/1)	N
PROPGL (0/1) ~ MI	N	PROPGL (0/1) ~ Placenta (0/1)	N
SDISL (0/1) ~ MI	Y	SDISL (0/1) ~ Placenta (0/1)	Y

Supplementary Table 14. Direct comparison of correlated evolution analyses in which the Matrotrophy Index is a continuous variable (phylogenetic logistic regression) and placentation is a binary character (BayesTraits Discrete analyses). The results of these two modelling efforts concord well. The only discrepancy is for analyses of courtship; with phylogenetic logistic regression the relationship approaches significance (*p*=0.0760) while in BayesTraits Discrete analysis, correlated evolution is supported (Bayes Factor = 4.192). In the present manuscript, we converted the Matrotrophy Index into a binary character (i.e. placenta presence or absence) so that we could go a step beyond testing for correlation, and make inferences regarding the order of evolution of male sexually selected traits and the placenta using BayesTraits Discrete models (which require 2 binary traits).

Model type	Df	lnLik	AIC	ChiSq	Pr(>|Chi|)	Significance
Constant λ	3	72.328	-138.660			
Linear λ	4	75.015	-142.030	5.374	0.02044	*
Sigmoidal λ	6	80.322	-148.640	15.988	0.00114	**
Modal λ	6	79.799	-147.600	14.942	0.00187	**
Linear λ, with directionality	5	75.227	-140.450	5.798	0.05508	.
Sigmoidal λ, with directionality	7	89.020	-164.040	33.383	0.00000	***
Modal λ, with directionality	7	88.184	-162.370	31.711	0.00000	***

Supplementary Table 15. Summary of QuaSSE model fits for the correlation between the Matrotrophy Index (MI) and speciation rate in poeciliid fishes. To evaluate the relationship between the Matrotrophy Index and speciation rate, we implemented QuaSSE (quantitative state speciation and extinction) models[8] in the R package diversitree[4]. To account for incomplete taxon sampling we specified the number of species with a Matrotrophy Index out of the total described species in the subfamily Poeciliinae (146 / 273). We compared the fit of alternative models in an ML framework using AIC and lnLik (by means of Chi-square test). Models tested included a Constant, Linear, Sigmoidal, or Modal relationship between the Matrotrophy Index and Speciation rate (λ). The Linear, Sigmoidal, and Modal models were also fit including a term allowing for directional evolution of the Matrotrophy Index. The sigmoidal model with directional evolution provided the best fit to the data. This model function is plotted in Supplementary Figure 22b.

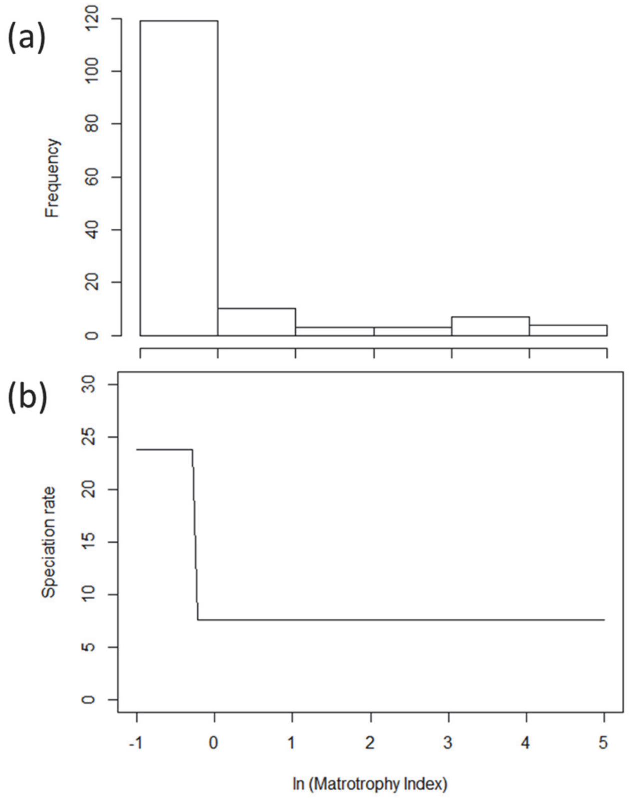

Supplementary Figure 22. Poeciliinae speciation rate as a function of the continuous Matrotrophy Index. (a) Frequency distribution of ln (Matrotrophy Index) for species in the subfamily Poeciliinae. (b) Best-fitting QuaSSE model relating speciation rate and ln (Matrotrophy Index). The best fitting ML speciation rate model was a sigmoidal function with directionality (Supplementary Table 15). This model shows a higher speciation rate in lecithotrophic species (l.y0=23.82) and lower speciation rate in matrotrophic species (l.y1=7.61) with an inflection point (l.xmid) at -0.23, which corresponds to a Matrotrophy Index of 0.80, and a high steepness of the sigmoid (l.r=18516.89).

According to the best-fitting QuaSSE model, species with a Matrotrophy Index less than 0.80 (i.e. Ln (MI) = -0.23) exhibit a substantially higher speciation rate than species with a Matrotrophy Index greater than 0.80. The inflection point of 0.80 approximates the cutoff between lecithotrophic and matrotrophic species. This lends support for our binary categorization of species as placental or non-placental. Overall, the best-fitting QuaSSE model supports interpretations derived from our BiSSE analysis in which we tested for a correlation between speciation rate and presence or absence of the placenta (Figure 5, Supplementary Figure 19, Supplementary Table 9). The BiSSE model also indicated that lecithotrophic species (i.e. non-placental) exhibited higher speciation rate than placental species, although Bayesian analyses indicated that the 95% credibility intervals did not differ (Figure 5 and Supplementary Figure 19). The best-fitting QuaSSE model also included a positive directionality parameter (drift=6.64) indicating a directional component to character evolution (i.e. matrotrophy) over time. In other words, the model picked up a signal of directional change toward increasing Matrotrophy Index (i.e. lecithotrophy to matrotrophy) over the course of evolution, which could be due to selection or any other within lineage process. This is consistent with our findings from ancestral state reconstructions – numerous independent origins of the placenta and the placenta, once gained, doesn't appear to have been lost.

Supplementary References

1. Paradis E, Schliep K. ape 5.0: an environment for modern phylogenetics and evolutionary analyses in R. *Bioinformatics* **35**, 526-528 (2019).

2. Maddison WP, Maddison DR. Mesquite: a modular system for evolutionary analysis. Version 3.51. http://mesquiteproject.org (2018).

3. Rabosky DL, Goldberg EE. FiSSE: A simple nonparametric test for the effects of a binary character on lineage diversification rates. *Evolution* **71**, 1432-1442 (2017).

4. FitzJohn RG. Diversitree: comparative phylogenetic analyses of diversification in R. *Methods in Ecology and Evolution* **3**, 1084-1092 (2012).

5. Revell LJ. Phytools: an R package for phylogenetic comparative biology (and other things). *Methods in Ecology and Evolution* **3**, 217-223 (2012).

6. Ives AR, Garland Jr T. Phylogenetic logistic regression for binary dependent variables. *Systematic Biology* **59**, 9-26 (2010).

7. Ho LsT, Ané C. A linear-time algorithm for Gaussian and non-Gaussian trait evolution models. *Systematic Biology* **63**, 397-408 (2014).

8. FitzJohn RG. Quantitative traits and diversification. *Systematic Biology* **59**, 619-633 (2010).
